# Synthesis of monophosphorylated lipid A precursors using 2-naphthylmethyl ether as a protecting group

**DOI:** 10.3762/bjoc.16.162

**Published:** 2020-08-10

**Authors:** Jundi Xue, Ziyi Han, Gen Li, Khalisha A Emmanuel, Cynthia L McManus, Qiang Sui, Dongmian Ge, Qi Gao, Li Cai

**Affiliations:** 1 Shanghai University of Engineering Science, 333 Long Teng Road, Shanghai 201620, China; 2 China State Institute of Pharmaceutical Industry, 285 Gebaini Rd, Shanghai 201203, China; 3 Department of Chemistry, University of South Carolina Lancaster, 476 Hubbard Drive, Lancaster, South Carolina 29720, USA,; 4 Suzhou Jingye Medicine & Chemical Co., Ltd, 88 Sanlian Street, Suzhou, Jiangsu Province, 215129, China

**Keywords:** lipid A, lipid X, lipopolysaccharide, 2-naphthylmethyl ether, synthesis

## Abstract

Lipid A, the hydrophobic domain of lipopolysaccharide (LPS), is a strong immunostimulator and therefore a valuable target for the development of novel immunomodulators. Various lipid A derivatives have been chemically synthesized in order to reduce toxicity while retaining the immunostimulatory activity. In this work, we describe a novel approach to the frequently problematic synthesis of monophosphorylated mono- and disaccharide lipid X using a combination of established chemistry and a novel 2-naphthylmethyl ether (Nap) protecting group for “permanent” protection of hydroxy groups. Of particular note is the fact that the key Nap protecting group is able to remain in the molecule until the final global deprotection step. Our synthetic strategy is not only efficient in regards to the yield of the various chemical transformations, but also robust in regards to the potential application of this route to the production of other lipid A analogs.

## Introduction

**Figure 1:**
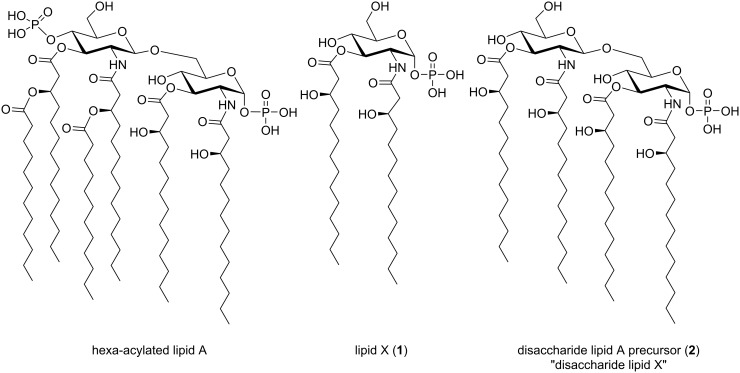
Chemical structures of hexa-acylated *Escherichia coli* lipid A, monophosphorylated lipid X (the reducing monosaccharide lipid A precursor), and disaccharide lipid A precursor (“disaccharide lipid X”).

Bacterial cell surfaces are decorated with various types of glycoconjugates (in the form of glycoproteins and glycolipids) that are known to participate in many biological processes, especially in the interactions between bacteria and the environment [1]. For example, lipopolysaccharide (LPS) comprises the Gram-negative bacterial cell wall and is crucial in bacterial pathogenicity [2]. LPS is a complex molecule that is composed of three structural regions: lipid A (endotoxin), a non-repeating core oligosaccharide, and *O*-antigen [2]. While *O*-antigen and the core oligosaccharide are exposed to the external environment, lipid A, the hydrophobic domain of LPS, is embedded in the cell wall. The lipid A substructure is relatively conserved that consists of a β-1,6-linked diglucosamine with 1,4′-di-*O*-phosphorylation and 2,2′-*N*- and 3,3′-*O*-acylation (Figure 1). The associated fatty acid acyl chains may be conserved within a species but can vary significantly in terms of the chain number and length for lipid A of different bacterial origins [3–4]. Lipid A represents a particularly important subject to research given the continued rise of problematic bacterial infections. Notably, the LPS pathogenicity is almost entirely due to lipid A because it leads to immunostimulatory effects when LPS dissociates from bacterial membranes within a host [5]. While these immunostimulatory effects can be beneficial in the setting of localized infections, the occurrence of severe sepsis causes systemic release of inflammation mediators and stimulatory molecules, thus leading to various pathophysiological effects [6]. Accordingly, structure–activity relationship studies of lipid A which examine or facilitate the examination of how one might harness these immunostimulatory effects are particularly valuable as they can provide basis for the development of vaccines and adjuvants. For example, recent studies have disclosed that both the fatty acid structure and the phosphorylation degree can affect the activity and endotoxic effects [7–9].

Various lipid A derivatives have since been synthesized to dissociate endotoxic effects from beneficial immunomodulatory activities. Lipid X, 2-*N*;3-*O*-di[(*R*)-3-hydroxytetradecanoyl]-ᴅ-glucosamine-1-phosphate, is the naturally occurring early monosaccharide precursor of lipid A biosynthesis (structure **1**, Figure 1). It was found that lipid X retained some immunomodulatory activity while having drastically reduced toxicity [10–11]. Lipid X was also found to give partial protection against a 100% lethal dose of endotoxin in mice [11]. However, there were also studies with conflicting results that showed that synthetic lipid X could be contaminated with small amounts of disaccharide-1-phosphate containing four (*R*)-3-hydroxytetradecanoic acids at the 2,2’ and 3,3’ positions (structure **2**, Figure 1). This disaccharide precursor **2** was identified as the main immunostimulatory side product [12–13]. While the research suggested chemically pure lipid X had no immunostimulatory properties of lipid A, it did behave as a competitive inhibitor of LPS [13].

In this paper we describe a synthesis of lipid X (**1**) and the disaccharide lipid A precursor **2** (2,2′-*N*;3,3′-*O-*tetra[(*R*)-3-hydroxytetradecanoyl]-β(1→6)-ᴅ-glucosamine disaccharide 1-phosphate) (Figure 1). The synthesis of such precursors is particularly important as it will facilitate the aforementioned goal of harnessing the immunostimulatory effects of lipid A through development of a clear understanding of the structure–activity relationship. More importantly, we employed the 2-naphthylmethyl ether (Nap) group for protection of various hydroxy groups on the carbohydrate and acyl moieties, aiming to provide an advantage over previous methods that mainly used the benzyl group [4,14–16] in synthesizing lipid A derivatives. We also aim at developing a robust strategy in regards to the potential application of our route to the production of other lipid A analogs.

## Results and Discussion

**Scheme 1:**
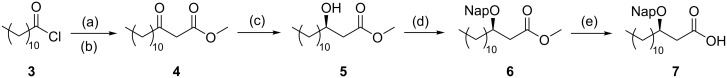
Enantioselective synthesis of Nap-protected (*R*)-3-hydroxytetradecanoic acid (**7**). Conditions: (a) Meldrum's acid, pyridine, CH_2_Cl_2_, 0 °C; (b) CH_3_OH, reflux, 77% over two steps; (c) (*R*)-Ru(OAc)_2_(BINAP), H_2_, CH_3_OH, 65 °C, 98%; (d) NapCHO, TMSOTf, (TMS)_2_O, Et_3_SiH, THF, 0 °C; (e) LiOH, THF, H_2_O, 65 °C, 78% (over two steps).

The acyl chain (*R*)-3-(2-naphthylmethoxy)tetradecanoic acid **7** was prepared via an enantioselective route as previously reported (Scheme 1) [16]. Lauroyl chloride (**3**) was treated with Meldrum’s acid (2,2-dimethyl-1,3-dioxane-4,6-dione) followed by decarboxylation in methanol to give methyl 3-oxotetradecanoate (**4**) in 77% yield. The enantioselective hydrogenation of the β-carbonyl group using (*R*)-Ru(OAc)_2_(BINAP) at 65 °C and under 1.5 MPa H_2_ afforded methyl (*R*)-3-hydroxytetradecanoate (**5**) in 98% yield. The same hydrogenation reaction was carried out using the (*S*)-Ru(OAc)_2_(BINAP) catalyst. Then both the *R* and *S* products were compared using chiral HPLC to confirm the absolute configuration and enantiomeric purity (Figure S1, Supporting Information File 1). The 3-hydroxy group in **5** was then protected as a Nap ether through a TMSOTf-catalyzed one-pot reductive naphthylmethylation process [17–18], by which free hydroxy groups were first trimethylsilylated in situ with hexamethyldisiloxane ((TMS)_2_O) before being naphthylmethylated by treatment with 2-naphthaldehyde, trimethylsilyl trifluoromethanesulfonate (TMSOTf), and triethylsilane (Et_3_SiH) [17,19]. On a 10 g scale, the protected methyl ester **6** could be purified by recrystallization followed by filtration to remove the major byproduct 2-methylnaphthalene. Subsequent saponification of the methyl ester **6** with LiOH gave (*R*)-3-(2-naphthylmethoxy)tetradecanoic acid (**7**) in 78% yield over two steps.

**Scheme 2:**
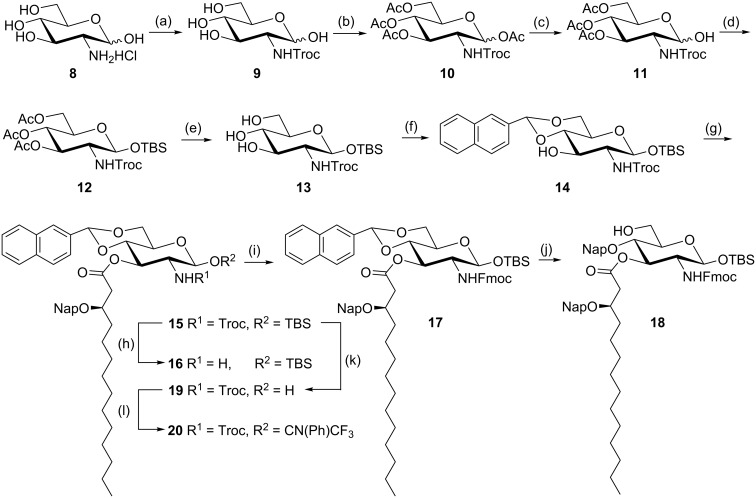
Synthesis of monoacylated glucosamine building blocks. Conditions: (a) NaHCO_3_, TrocCl, H_2_O, 0 °C, 94% ; (b) Ac_2_O, pyridine, rt, 96%; (c) N_2_H_4_, AcOH, DMF, rt, 89%; (d) TBSCl, imidazole, DMF, rt, 93%; (e) guanidine hydrochloride buffer, rt; (f) NapC(OMe)_2_, camphorsulfonic acid, CH_3_CN, rt, 68% (2 steps); (g) acid **7**, EDC·HCl, DMAP, CH_2_Cl_2_, rt, 85%; (h) Zn/AcOH, CH_2_Cl_2_, rt; (i) DIPEA, FmocCl, CH_2_Cl_2_, rt, 80% (2 steps); (j) PhBCl_2_, Et_3_SiH, CH_2_Cl_2_, MS 4 Å, −78 °C, 80%; (k) HF/pyridine, THF,−40 °C to rt, 93%; (l) DBU, ClCN(Ph)CF_3_, CH_2_Cl_2_, 95%.

The glucosamine building block **14** was synthesized using the procedures described in previous literature [20] (Scheme 2). The protection of the free amine of glucosamine with a 2,2,2-trichloroethoxycarbonyl (Troc) group under basic conditions followed by peracetylation afforded compound **10** on a ≈150 g scale. The regioselective anomeric deacetylation with hydrazine and reprotection of the anomeric hydroxy group as *tert*-butyldimethylsilyl ether (TBS) led to compound **12**. Compound **12** was then treated with sodium methoxide in guanidine hydrochloride buffer solution (pH ≈ 9) to remove the *O*-3,4,6-acetyl groups [14]. Because the deacetylation reaction was later neutralized with cation exchange resin, extra washing with saturated NaHCO_3_ during reaction work-up seemed necessary to avoid cleavage of the TBS ether in compound **13**. Then, (2-naphthyl)methylene acetal [21] was used to protect the C-4,6-hydroxy groups using 2-naphthaldehyde dimethyl acetal and 0.2 equiv of camphorsulfonic acid (CSA). These protecting group manipulations resulted in the exposure of the C-3 hydroxy group in compound **14** for further acylation [4]. They are also essential for orthogonal protection of glucosamine, allowing the specific deprotection in subsequent steps (for example, the arylidene acetals at O4 and O6 could be regioselectively opened and transformed into Nap ethers) [19]. The C-3 hydroxy group in compound **14** was then acylated with (*R*)-3-(2-naphthylmethoxy)tetradecanoic acid (**7**) using 1-ethyl-3-(3-dimethylaminopropyl)carbodiimide (EDC) and 4-dimethylaminopyridine (DMAP) as the activation reagents [14] to give the key/common building block **15** in good yield (Scheme 2).

Glycosyl acceptor **18** and donor **20** were thus conveniently prepared from the common building block **15** through multiple protecting group manipulations (Scheme 2). The *N*-Troc group in **15** was removed by treatment with zinc in a mixture of acetic acid and CH_2_Cl_2_. The resulting amine **16** was protected immediately as fluorenylmethylenoxy (Fmoc) carbamate by reaction with FmocCl in the presence of diisopropylethylamine (DIPEA) to give the fully protected compound **17**. The regioselective opening of the arylidene acetal at O6 with Et_3_SiH and PhBCl_2_ in the presence of molecular sieves at −78 °C [22] gave compound **18** in good yield (80%) having a free C-6 hydroxy group. Compound **18** is the glycosyl acceptor for the synthesis of the disaccharide lipid A precursor (Scheme 4). For the synthesis of donor **20**, first removal of the anomeric TBS in building block **15** was achieved by treatment with HF-pyridine followed by conversion of the resulting lactol into the desired *N*-phenyltrifluoroacetimidate glycosyl donor **20** by reaction with 2,2,2-trifluoro-*N*-phenylacetimidoyl chloride in the presence of base DBU [14].

**Scheme 3:**
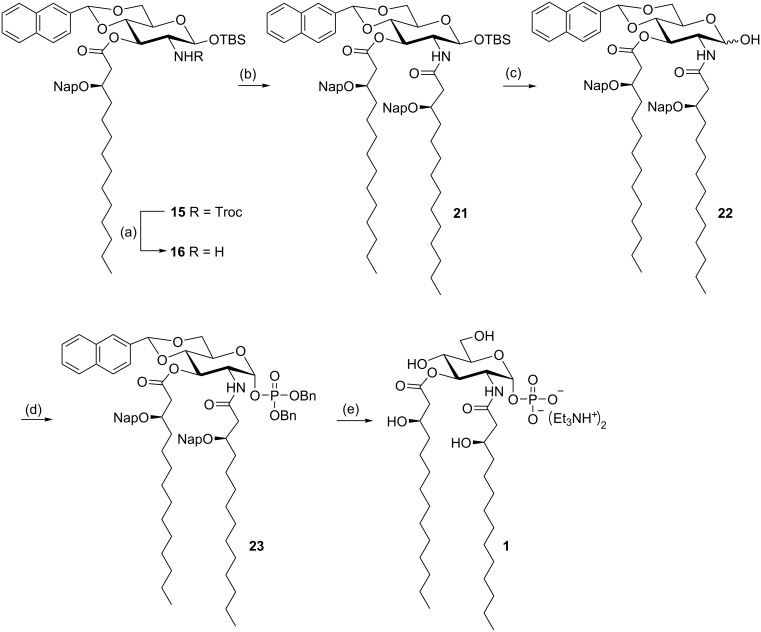
Synthesis of lipid X monosaccharide **1**. Conditions: (a) Zn, AcOH, CH_2_Cl_2_, rt; (b) acid **7**, EDC·HCl, DMAP, CH_2_Cl_2_, rt, 67.5% (2 steps); (c) HF/Py, THF, −40 °C to rt, 78%; (d) tetrabenzyl pyrophosphate, LHMDS, THF, −78 °C, 91%; (e) H_2_ (15 kg/cm^2^), Pd/C, THF/H_2_O, 38 °C, 86%.

The monoacylated derivative **15** is also the key building block for the synthesis of lipid X monosaccharide **1** (Scheme 3). After the *N*-Troc protecting group was removed as described above, the free amine was immediately acylated with (*R*)-3-(2-naphthylmethoxy)tetradecanoic acid (**7**) using EDC and DMAP as the activation reagents to give the diacylated compound **21** in good yield. The anomeric TBS ether of **21** was then cleaved with HF, and the resulting anomeric hydroxy group was phosphorylated using tetrabenzyl diphosphate in the presence of lithium bis(trimethyl)silylamide (LHMDS) in THF at −78 °C [23] to afford the anomeric phosphate **23** exclusively as the α-anomer. Finally, global deprotection of **23** (benzyl phosphate, Nap ethers, and naphthylidene acetal) were accomplished by catalytic hydrogenolysis over Pd/C under 15 kg/cm^2^ of H_2_ to give the target lipid X monosaccharide **1** (as triethylammonium salt) in good yield.

**Scheme 4:**
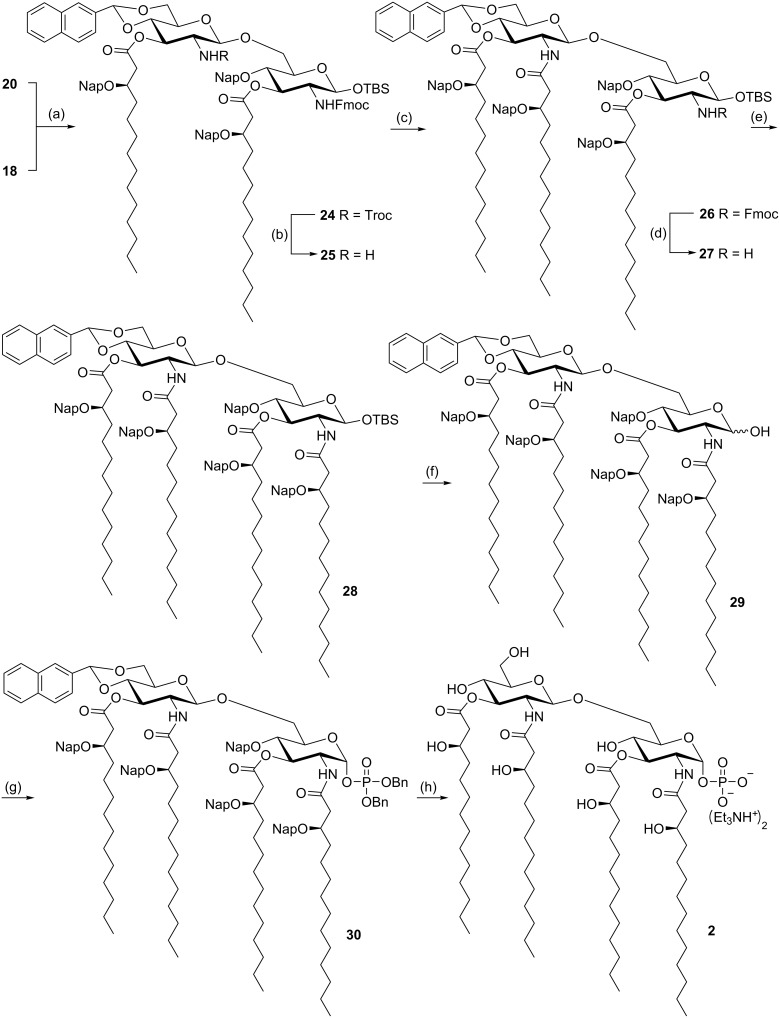
Synthesis of the disaccharide lipid A precursor **2**. Conditions: (a) TfOH, 4 Å MS, dry CH_2_Cl_2_, 94%; (b) Zn, AcOH, CH_2_Cl_2_; (c) acid **7**, EDC·HCl, DMAP, CH_2_Cl_2_, 88% (2 steps); (d) Et_3_N, DMF; (e) acid **7**, EDC·HCl, CH_2_Cl_2_, 82% (2 steps); (f) HF/pyridine, pyridine, THF, −40 °C to rt, 92%; (g) tetrabenzyl pyrophosphate, LHMDS, dry THF, −78 °C, 82%; (h) H_2_, Pd-black, THF, 38 °C, 88%.

Having the glycosyl donor **20** and acceptor **18** at hand (Scheme 2), in order to prepare the disaccharide precursor, the glycosylation reaction was performed first, followed by deprotection, acylation, and phosphorylation reactions (Scheme 4). The triflic acid (TfOH)-mediated glycosylation of donor **20** and acceptor **18** in the presence of molecular sieves in CH_2_Cl_2_ at −20 °C gave disaccharide **24** [14] in excellent yield (β-anomer only). The *N’*-Troc protecting group (non-reducing end) was first removed using Zn dust in acetic acid, and the resulting free amine was immediately acylated with (*R*)-3-(2-naphthylmethoxy)tetradecanoic acid (**7**) using EDC and DMAP as the coupling reagents to afford triacylated disaccharide **26**. Then, the *N*-Fmoc group (reducing end) in **26** was removed by treatment with triethylamine, and the resulting amine again was immediately acylated with (*R*)-3-(2-naphthylmethoxy)tetradecanoic acid (**7**) to afford disaccharide **28** with four fatty acid chains. After cleavage of the anomeric TBS moiety employing HF in pyridine, the resulting anomeric hydroxy group of **29** was phosphorylated using tetrabenzyl diphosphate in the presence of LHMDS in THF at −78 °C. Then finally global deprotection (hydrogenation over Pd-black) was carried out to remove the naphthylidene acetal, Nap ethers, and the benzyl phosphate groups in compound **30**. By this route the target disaccharide lipid A precursor **2** (as triethylammonium salt) was obtained in 88% yield.

## Conclusion

As described, we have developed an efficient approach for the chemical synthesis of two monophosphorylated lipid A precursors. Lipid X (**1**) could be prepared from the common building block **15** via deprotection, acylation, phosphorylation, and global deprotection. The glycosyl acceptor and donor for the synthesis of the disaccharide precursor could also be readily obtained starting from the same key building block. After glycosylation, the disaccharide lipid A precursor **2** was synthesized following a similar reaction sequence of deprotection, acylation, phosphorylation, and global deprotection.

The Nap protecting group has emerged as a particularly valuable addition to carbohydrate chemistry [24–25]. Not only does it not significantly alter carbohydrate reactivity, it also can be readily cleaved under hydrogenolytic conditions as well as a variety of oxidative [26] and acid-mediated conditions [25,27] that are orthogonal to benzyl ethers. Therefore, we employed the Nap ether as a “permanent” protecting group for the carbohydrate and the 3-hydroxy group of the acyl chain, aiming to provide an advantage over literature reported methods that mainly used the benzyl group in synthesizing lipid A derivatives. Of particular note is the fact that the key Nap protecting group is able to remain in the molecule until the final global deprotection step. The presence of this protecting group until such a late stage likely helps avoiding the problematic acyl migration often observed with similar molecules [28]. In addition, the 4,6-*O*-naphthylidene acetal (e.g., in compounds **23** and **30**) can be regioselectively opened at O6 or O4 under different conditions [19,29]. This could potentially allow the incorporation of other functionalities in target molecules for the synthesis of glycoconjugates. Based on the synthetic strategy described in this work (a common building block and Nap ether protection), we have already designed a route to MPLA, a clinically safe [30] monophosphoryl lipid A derivative with one phosphate group linked to the 4′-OH group. This work is currently underway in our lab.

## Supporting Information

File 1 Experimental details and copies of NMR spectra.
