# Supplementary material for: Synthesis of monophosphorylated lipid A precursors using 2-naphthylmethyl ether as a protecting group
Source: Beilstein J Org Chem. 2020 Aug 10;16:1955–62. doi: 10.3762/bjoc.16.162 (PMC7431767; doi:10.3762/bjoc.16.162)
Supplement: File 1 — Experimental details and copies of NMR spectra. [file Beilstein_J_Org_Chem-16-1955-s001.pdf]

BEILSTEIN JOURNAL OF ORGANIC CHEMISTRY

Supporting Information

for

Synthesis of monophosphorylated lipid A precursors using 2-naphthylmethyl ether as a protecting group

Jundi Xue, Ziyi Han, Gen Li, Khalisha A. Emmanuel, Cynthia L. McManus, Qiang Sui, Dongmian Ge, Qi Gao and Li Cai

Beilstein J. Org. Chem. **2020,** *16,* 1955–1962. doi:10.3762/bjoc.16.162

Experimental details and copies of NMR spectra

License and Terms: This is a supporting information file under the terms of the Creative Commons Attribution License (http://creativecommons.org/licenses/by/4.0). Please note that the reuse, redistribution and reproduction in particular requires that the authors and source are credited.
The license is subject to the *Beilstein Journal of Organic Chemistry* terms and conditions: (https://www.beilstein-journals.org/bjoc)

Table of contents page

Materials and methods S2

Synthetic procedure and analytical data..................... S2–S17

Figure S1: Chiral HPLC of compound **5** S18

References for known compounds..................... S18

NMR spectra for new compounds S19–S37

Materials and methods

Unless indicated, all commercial reagents were used as received without further purification. All reaction solvents were purified before use. Column chromatography was performed on silica gel, AR (200–300 mesh, General-Reagent). The enantiomeric excesses (ee) of the chiral β-hydroxy esters were determined with a Shimadzu/DGU-20A$_5$ HPLC apparatus fitted with a Chiralcel OD (0.46 × 25 cm) chiral column. Hexane and isopropanol (98:2 (v/v)) was used as the mobile phase, flow rate: 1.0 mL/min, wavelength: 210 nm. Reactions were monitored by thin-layer chromatography (TLC) on HSGF254; and the compounds were detected by examination under UV light and spots were visualized by spraying with a molybdate solution [0.02 M solution of ammonium cerium(IV) sulfate dihydrate and ammonium molybdate(VI) tetrahydrate in aq. H_2SO_4]. Concentration of solutions was performed at reduced pressure at temperatures < 30 °C. NMR spectra were recorded at 297 K in $CDCl_3$ (unless stated otherwise) with a Bruker Avance III HD 400 MHz and Bruker Avance NEO 600 MHz. ^{1}H NMR spectra were referenced to tetramethylsilane. ^{13}C NMR spectra were referenced to chloroform (δ 77.00 ppm). HRESIMS spectra were recorded on 6210 TOF (Agilent). ESIMS spectra were recorded on a Q-Tof micro (Waters) instrument.

Compound 4

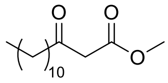

To a solution of Meldrum's acid (65.6 g, 0.3 mol) and pyridine (48 mL) in CH_2Cl_2 (100 mL) was added lauroyl chloride (**3**, 65.6 g, 0.3 mol) at 0 °C. The mixture was stirred at room temperature for 2.5 h. After complete consumption of the starting material as shown by TLC, the mixture was washed with 1 M HCl (100 mL) and water (100 mL). The organic layer was dried (anhydrous Na_2SO_4), filtered and the filtrate was concentrated in vacuo. Then, methanol (250 mL) was added to the residue and the resulting mixture was refluxed overnight. The mixture was then concentrated and purified by Al_2O_3 column chromatography (toluene/ethyl acetate 2:1) to give the methyl ester **4** as colorless thick syrup (59.3 g, 77%). ^{1}H NMR (400 MHz, $CDCl_3$) δ 3.70 (3 H, d, *J* = 1.5), 3.42 (2 H, s), 2.50 (2 H, t, *J* = 7.4), 1.28 (18 H, s), 0.90 (3 H, t, *J* = 6.6); ^{13}C NMR (101 MHz, $CDCl_3$) δ 202.51, 167.58, 137.67, 128.96, 128.15, 125.25, 52.02, 48.84, 42.90, 31.92, 29.62, 29.47, 29.38, 29.35, 29.01, 23.43, 22.68, 21.31, 14.04. Ref. [1].

Compound 5

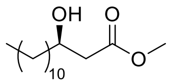

Compound **4** (15 g) and ruthenium catalyst (180 mg) (preparation of the ruthenium catalyst: to a solution of (*R*)-Ru(OAc)$_2$(BINAP) in CH_2Cl_2 (5 mL) was added 1.42 N HCl (0.35 mL), the mixture was then stirred at room temperature for 1 h and then concentrated in vacuo) were dissolved in methanol (50 mL). The resulting mixture was placed under H_2 (1.5 MPa) and stirred at 65 °C for 12 h. After complete consumption of the starting material as shown by TLC, the reaction mixture was concentrated and

purified by silica gel column chromatography (petroleum ether/ethyl acetate 5:1) to give compound **5** as white solid (15 g, 98%). ^{1}H NMR (400 MHz, $CDCl_3$) δ 4.01 (1 H, dq, *J* = 11.8, 4.0), 3.71 (3 H, s), 2.90 (1 H, d, *J* = 4.0), 2.46 (2 H, ddd, *J* = 25.4, 16.4, 6.1), 1.26 (18 H, s), 0.88 (3 H, t, *J* = 6.8); ^{13}C NMR (101 MHz, $CDCl_3$) δ 173.41, 67.99, 51.63, 41.18, 36.57, 31.89, 29.60, 29.56, 29.51, 29.32, 25.47, 22.65, 14.05. Ref. [1].

Compound 7 ((*R*)-3-naphthyloxytetradecanoic acid)

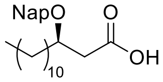

To a solution of **5** (10 g, 39 mmol) and NapCHO (18.14 g, 116 mmol) in THF (100 mL) were added TMSOTf (6.88 g, 31 mmol), $(TMS)_2O$ (37.68 g, 232 mmol), and Et_3SiH (15.7 g, 135 mmol) at 0 °C. The mixture was stirred for 1.5 h at 0 °C and then allowed to warm to room temperature (the 2-methylnaphthalene byproduct may precipitate from the reaction mixture). The reaction mixture was filtered and then diluted with CH_2Cl_2 (150 mL) and washed with sat. aq. $NaHCO_3$. The organic layer was concentrated in vacuo and the residue was recrystallized from petroleum ether/ethyl acetate 5:1, and filtered to further remove impurities (2-methylnaphthalene). The filtrate was collected and concentrated in vacuo to afford the crude product **6** as yellowish solid which was used directly in the next step without further purification. A solution of **6** in THF/H_2O 5:1 (100 mL) was vigorously stirred with an aqueous solution of lithium hydroxide (9.41 g, 224 mmol, 94 mL) under reflux for 2 h. After complete consumption of the starting material as shown by TLC, the mixture was cooled to room temperature and quenched by the addition of 1.5 M aq. HCl to a pH value of 7. The mixture was then diluted with CH_2Cl_2 (150 mL), washed with sat. aq. $NaHCO_3$ (150 mL), dried (anhydrous Na_2SO_4) and concentrated in vacuo. The residue was purified by silica gel column chromatography (petroleum ether/ethyl acetate 5:1) to afford compound **7** as colorless thick syrup (11.6 g, 77.9% over two steps). ^{1}H NMR (400 MHz, $CDCl_3$) δ 10.00 (1 H, s, OH), 7.85 (4 H, dd, *J* = 14.5, 10.8), 7.53 (3 H, d, *J* = 4.2), 4.85 – 4.71 (2 H, m, $NapCH_2O$), 4.08 – 3.94 (1 H, m, H-3), 2.76 (1 H, dd, *J* = 15.2, 6.9, H-2), 2.64 (1 H, dd, *J* = 15.2, 4.3, H-2), 1.84 – 1.58 (2 H, m, H-4), 1.57 –1.31 (18 H, m), 1.00 (3 H, t, *J* = 6.0); ^{13}C NMR (101 MHz, $CDCl_3$) δ 177.86, 135.86, 133.38, 133.10, 128.20, 128.00, 127.76, 126.57, 126.10, 125.99, 125.89, 75.94, 71.71, 39.81, 34.37, 32.05, 29.77, 29.71, 29.48, 25.27, 22.82, 14.26. HRESI-MS calcd. for $C_{25}H_{36}O_3Na$ ($[M+Na]^+$): 407.2557, found 407.2552 *m/z*.

Compound 9

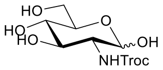

To a solution of glucosamine hydrochloride **8** (85.53 g, 397 mmol) and $NaHCO_3$ (83.3 g, 992 mmol) in H_2O (300 mL) was added TrocCl (126.1 g, 595 mmol) at 0 °C. The reaction mixture was stirred for 1 h. After complete consumption of the starting material as shown by TLC, the reaction mixture was filtered and the filtrate was washed by large amounts of H_2O and petroleum ether to give compound **9** as white solid (132.1 g, 94%). ^{1}H NMR (400 MHz, CD_3OD) δ 5.16 (1 H, d, *J* = 3.4, H-1), 4.85–4.71 (2 H, m, OCH_2CCl_3), 3.82 (2 H, dt, *J* = 5.9, 2.3, H-6), 3.75 – 3.68 (2H, m, H-2, H-3), 3.60 (1 H, dd, *J* = 10.6, 3.4, H-5), 3.39 (1 H, t, *J* = 9.2, H-4); ^{13}C NMR (101 MHz, CD_3OD) δ 155.51 (NHTroc), 95.73 (C-1), 91.39 (OCH_2CCl_3), 74.23 (OCH_2CCl_3), 71.69 (C-5), 71.31 (C-3), 71.02 (C-4), 61.42 (C-6), 56.48 (C-2). Ref. [2].

Compound 10

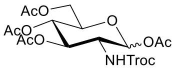

To a solution of **9** (102 g, 287 mmol) in pyridine (500 mL) was gradually added acetic anhydride (300 mL) at 0 °C. Then, the reaction was allowed to warm to room temperature and stirred overnight. After complete consumption of the starting material as shown by TLC, the reaction mixture was diluted with CH_2Cl_2 (300 mL) and washed with 2 M HCl (300 mL). The organic layer was dried (anhydrous Na_2SO_4), filtered and the filtrate was concentrated in vacuo to give compound **10** as yellowish syrup (144.7 g, 96.3%). ^{1}H NMR (400 MHz, $CDCl_3$) δ 6.24 (1 H, d, *J* = 3.6, H-1), 5.36 (1 H, d, *J* = 9.4, H-3), 5.28 (1 H, d, *J* = 10.6, H-4), 5.20 (1 H, t, *J* = 9.7, NH), 4.83 (1 H, d, *J* = 12.1, OCH_2CCl_3), 4.63 (1 H, d, *J* = 12.1, OCH_2CCl_3), 4.28 (1 H, dd, *J* = 12.2, 3.9, H-6), 4.21 (1 H, ddd, *J* = 10.7, 9.7, 3.7, H-2), 4.10 – 4.02 (2 H, m, H-6, H-5), 2.20 (3 H, s), 2.09 (3H, s), 2.05 (6 H, d, *J* = 3.0); ^{13}C NMR (101 MHz, $CDCl_3$) δ 171.17, 170.62, 169.16, 168.63, 154.10 (NHTroc), 95.25 (C-1), 90.40 (OCH_2CCl_3), 74.61 (OCH_2CCl_3), 70.36 (C-5), 69.69 (C-3), 67.61 (C-4), 61.50 (C-6), 53.18 (C-2), 20.85, 20.64, 20.62, 20.52. Ref. [2].

Compound 11

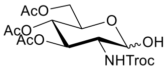

To solution of compound **10** (150 g, 287 mmol) in DMF (300 mL) were added hydrazine hydrate (18.7 g, 0.374 mol) and acetic acid (23 mL, 0.39 mol) with cooling in an ice bath. Then, the reaction mixture was stirred at room temperature for 0.5 h. After complete consumption of the starting material as shown by TLC, the mixture was diluted with CH_2Cl_2 (450 mL × 2) and washed with H_2O (450 mL). The organic layer was dried (anhydrous Na_2SO_4), filtered and the filtrate was concentrated in vacuo to give compound **11** as colorless syrup (123 g, 89.2%). ^{1}H NMR (400 MHz, $CDCl_3$) δ 5.46 (1 H, d, *J* = 9.8, NH), 5.38 – 5.27 (2 H, m, H-1, H-3), 5.17 – 5.09 (1 H, m, H-4), 4.80 (1 H, d, *J* = 12.0, OCH_2CCl_3), 4.64 (1 H, d, *J* = 12.0, OCH_2CCl_3), 4.27 – 4.20 (2 H, m, H-6) , 4.17–4.11 (1 H, m, H-5), 4.09 – 4.02 (1 H, m, H-2), 2.11 (3 H, s),

2.04 (3 H, s), 2.02 (3 H, s); ^{13}C NMR (101 MHz, $CDCl_3$) δ 171.04, 170.88, 169.50, 154.26, 95.37 (C-1), 91.81 (OCH_2CCl_3), 74.62 (OCH_2CCl_3), 70.76 (C-5), 68.31 (C-3), 67.74 (C-4), 62.05 (C-6), 54.20 (C-2), 20.79, 20.71, 20.64. Ref. [2].

Compound 12

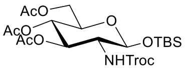

To a solution of compound **11 (**138 g, 287 mmol) and imidazole (78.2 g, 1.15 mol) in DMF (200 mL), was added TBSCl (151.4 g, 1 mol) with cooling in an ice bath. Then, the mixture was stirred for 12 h at room temperature. After complete consumption of the starting material as shown by TLC, H_2O (1000 mL) was added directly and the mixture stirred for another hour to precipitate the solid. The residue was further purified by recrystallization (petroleum ether/ethyl acetate 9:2) to give compound **12** as white solid (158 g, 92.9 %). ^{1}H NMR (400 MHz, $CDCl_3$) δ 5.28 – 5.21 (2 H, m, H-1, H-3), 5.03 (1 H, t, *J* = 9.6, H-4), 4.81 (1 H, d, *J* = 7.8, NH), 4.74 (1 H, d, *J* = 12.0, OCH_2CCl_3), 4.65 (1 H, d, *J* = 12.0, OCH_2CCl_3), 4.21 (1 H, dd, *J* = 12.1, 5.9, H-6), 4.13 (1 H, dd, *J* = 12.0, 2.3, H-6), 3.75 – 3.69 (1 H, m, H-5), 3.63 (1 H, dt, *J* = 10.6, 9.1, H-2), 2.05 (9 H, d, *J* = 18.5), 0.88 (9 H, s), 0.11 (6 H, d, *J* = 10.1); ^{13}C NMR (101 MHz, $CDCl_3$) δ 170.78, 170.62, 169.51, 154.02, 96.13, 95.32, 74.59, 71.96, 71.82, 69.13, 62.51, 58.22, 25.53, 20.69, 20.64, 17.90. Ref. [2].

Compound 14

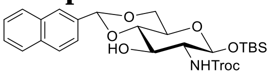

G·HCl buffer solution (100 mL, see below for preparation) was added slowly to compound **12 (**10 g, 16.8 mmol), and the reaction mixture was stirred at room temperature for 2 h. After complete consumption of the starting material as shown by TLC, the reaction mixture was neutralized with cation exchange resin (washed with methanol three times). Then, the organic layer was concentrated in vacuo, diluted with CH_2Cl_2 (40 mL), and washed with sat. aq. $NaHCO_3$ (30 mL). The organic layer was then concentrated to give compound **13** (8.23 g) as yellow solid which was used directly in the next step. To a solution of **13** (8.23 g) and $NapC(OMe)_2$ (5.1 g, 25.2 mmol) in CH_3CN (50 mL), was added camphorsulfonic acid (0.78 g, 3.36 mmol) at room temperature and the reaction mixture was stirred for 3 h. After complete consumption of the starting material as shown by TLC, the reaction was quenched by the addition of sat. aq. $NaHCO_3$ (30 mL) and diluted with CH_2Cl_2 (30 mL). The organic layer was dried (anhydrous Na_2SO_4), filtered and the filtrate was concentrated in vacuo. The residue was purified by silica gel column chromatography (petroleum ether/ethyl acetate 5:1) to give compound **14** as yellowish solid (6.97 g, 68.3% over two steps). ^{1}H NMR (400 MHz, $CDCl_3$) δ 7.96 (1 H, s), 7.89 – 7.80 (3 H, m), 7.60 (1 H, dd, *J* = 8.5, 1.5), 7.54 – 7.45 (2 H, m), 5.66 (1 H, s, CHNap), 5.13 (1 H, d, *J* = 8.2, NH), 4.73 (2 H, d, *J* = 8.0, H-1, OCH_2CCl_3), 4.66 (1 H, d, *J* = 11.9, OCH_2CCl_3), 4.30 (1 H, dd, *J* = 10.5, 5.0, H-6), 3.91 (1 H, br, H-3), 3.79 (1 H, t, *J* = 10.3, H-6), 3.57 (1 H, t, *J* = 9.2, H-4), 3.46 – 3.40 (1 H, m, H-5), 3.37 (1 H, dd, *J* = 15.5, 6.5, H-2), 3.04 (1H, s, OH） 0.88 (9 H, s), 0.10 (6 H, dd, *J* = 8.0, 3.0); ^{13}C NMR (101 MHz, $CDCl_3$) δ 154.54,

134.52, 133.78, 132.87, 128.41, 128.29, 127.75, 126.69, 126.41, 126.07, 123.93, 101.97, 96.33(C-1), 95.30, 81.52 (C-4), 74.85 (OCH_2CCl_3), 70.71 (C-3), 68.68 (C-6), 66.20 (C-5), 60.73 (C-2), 26.94, 25.59, 17.90, -4.14, -5.26. HRESI-MS calcd. for $C_{26}H_{34}Cl_3NNaO_7Si$ ($[M+Na]^+$): 628.1062, found 628.1075 *m/z*. Ref. [3].

Preparation of guanidine hydrochloride (G·HCl) buffer solution: guanidine hydrochloride (4.776 g) was dissolved in methanol (900 mL), and then Na (230 mg) and CH_2Cl_2 (100 mL) were to the mixture, pH ≈ 9.

Compound 15

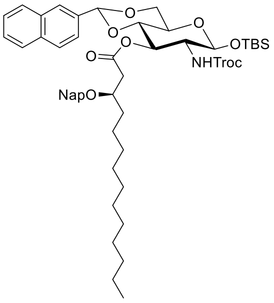

To a solution of compound **14** (3.78 g, 6.2 mmol) and (*R*)-3-naphthyloxytetradecanoic acid (**7**, 3.59 g, 9.3 mmol) in CH_2Cl_2 (35 mL) were added EDC·HCl (2.37 g, 12.4 mmol), and DMAP (0.15 g, 1.2 mmol) at 0 °C. Then, the reaction mixture was allowed to warm to room temperature and stirred for another 4 h. After complete consumption of the glucopyranoside starting material as shown by TLC, the reaction mixture was diluted with CH_2Cl_2 (30 mL) and washed with sat. aq. $NaHCO_3$ (30 mL). The organic phase was dried (anhydrous Na_2SO_4) and concentrated and the residue was purified by silica gel column chromatography (petroleum ether/ethyl acetate 10:1) to afford compound **15** as white solid (5.12 g, 85 %). ^{1}H NMR (400 MHz, $CDCl_3$) δ 7.81 (1 H, s), 7.76 – 7.61 (7 H, m), 7.48 – 7.35 (5 H, m), 7.30 (1 H, d, *J* = 8.4), 5.50 (1 H, s, NapCH), 5.39 (1 H, t, *J* = 9.9,H-3), 5.24 (1 H, d, *J* = 9.1, NH), 4.84 (1 H, d, *J* = 7.8, H-1), 4.67 – 4.62 (3 H, m, $NapCH_2$, $-OCH_2CCl_3$), 4.52 (1 H, d, *J* = 11.8, -OCH_2CCl_3), 4.31 (1 H, dd, *J* = 10.4, 4.7, H-6), 3.85 – 3.81 (1 H, m, lipid-H-3), 3.78 (1 H, d, *J* = 10.3, H-6), 3.71 (1 H, t, *J* = 9.5, H-4), 3.67 – 3.60 (1 H, m, H-2), 3.53 (1 H, td, *J* = 9.5, 5.1, H-5), 2.74 (1 H, dd, *J* = 14.9, 6.0, lipid-H-2), 2.55 (1 H, dd, *J* = 14.8, 5.6, lipid-H-2), 1.54 (2 H, m, lipid-H-4), 1.27 (18 H, s), 0.91 (12 H, s), 0.13 (6 H, d, *J* = 12.0); ^{13}C NMR (101 MHz, $CDCl_3$) δ 171.82, 154.36, 136.11, 134.41, 133.85, 133.41, 133.13, 132.99, 128.53, 128.28, 128.23, 128.11, 127.88, 127.85, 126.60, 126.51, 126.31, 126.20, 126.04, 125.96, 125.91, 123.85, 101.88, 97.14 (C-1), 79.18 (C-4), 75.73(C-6), 74.88, 71.39 (C-3), 71.28, 68.91 (C-6), 66.76 (C-5), 60.64, 59.29 (C-2), 39.77, 34.71, 32.15, 29.87, 29.86, 29.80, 29.77, 29.58, 25.74, 25.45, 22.92, 21.28, 18.08, 14.43, 14.37, -3.98, -5.08. HRESI-MS calcd. for $C_{51}H_{72}Cl_3N_2O_9Si$ ($[M + NH_4]^+$): 989.4067, found 989.4049 *m/z*; calcd. for $C_{51}H_{68}Cl_3NNaO_9Si$ ($[M+Na]^+$): 994.3621, found 994.3659 *m/z*.

Compound 17

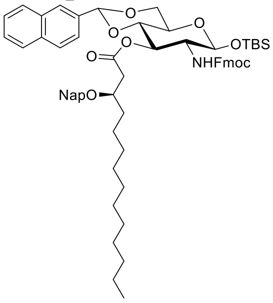

A solution of compound **15** (5 g, 5.1 mmol) in CH_2Cl_2 (50 mL) was placed under N_2 atmosphere, and Zn dust (5 g, 77 mmol) and acetic acid (10 mL) were added. After stirring at room temperature for 3 h, the reaction mixture was filtered and washed with sat. aq. $NaHCO_3$ (50 mL). The organic phase was dried (anhydrous Na_2SO_4) and concentrated. The deprotection of the Troc group (compound **16**) was confirmed by Q-TOF microMS analysis. The crude free amine **16** (4.4 g) was redissolved in CH_2Cl_2 (40 mL), and DIPEA (1.32 g, 10.2 mmol) and FmocCl (2.65 g, 10.2 mmol) were added at 0 °C. Then, the reaction mixture was allowed to warm to room temperature and stirred for another 2 h. Upon completion, the reaction mixture was diluted with CH_2Cl_2 (30 mL) and washed with sat. aq. NaCl (40 mL). The organic phase was dried (anhydrous Na_2SO_4) and concentrated and the residue was purified by crystallization (CH_2Cl_2/methanol 1:10) to afford compound **17** as white solid (4.2 g, 80%, two steps). ^{1}H NMR (400 MHz, $CDCl_3$) δ 7.83 (1 H, s), 7.78 – 7.65 (7 H, m), 7.64 – 7.44 (5 H, m), 7.40 (6 H, ddd, *J* = 15.3, 9.7, 5.3), 7.32 – 7.24 (3 H, m), 5.53 (1 H, s, NapCH), 5.44 (1 H, t, *J* = 9.4, H-3), 4.97 (1 H, d, *J* = 8.7, NH), 4.91 (1 H, d, *J* = 7.2, H-1), 4.63 (1 H, d, *J* = 11.8, Fmoc-CH_2), 4.51 (1 H, d, *J* = 11.8, , Fmoc-CH_2), 4.32 (1 H, br, H-6), 4.28 (2 H, d, *J* = 6.5, NapCH_2), 4.17 (1 H, d, *J* = 6.4, , Fmoc-CH), 3.82 (2 H, s, H-6, lipid-H-3), 3.78 – 3.71 (1 H, m, H-4), 3.67 (1 H, d, *J* = 8.9, H-2), 3.58 (1 H, br, H-5), 2.70 (1 H, dd, *J* = 14.8, 6.3), 2.50 (1 H, dd, *J* = 14.8, 5.5), 1.55 – 1.37 (2 H, m), 1.29 – 0.96 (18 H, m), 0.91 – 0.81 (12 H, m), 0.09 (6 H, d, *J* = 13.5); ^{13}C NMR (101 MHz, $CDCl_3$) δ 155.78, 143.79, 141.23, 135.92, 134.24, 133.63, 133.17, 132.87, 132.78, 128.31, 128.05, 127.92, 127.86, 127.63, 127.04, 126.34, 126.26, 126.06, 125.93, 125.81, 125.69, 125.15, 123.64, 119.96, 101.65, 97.14 (H-1), 79.00 (H-4), 75.62 (H-6), 71.25 (H-3),71.19, 68.76 (H-6), 67.19, 66.60 (H-5), 58.98 (H-2), 47.04, 39.74, 34.53, 31.92, 29.61, 29.53, 29.34, 25.49, 25.16, 22.69, 17.86, 14.14, -4.21, -5.36. HRESI-MS calcd. for $C_{63}H_{81}N_2O_9Si$ ($[M+NH_4]^+$): 1037.5706, found 1037.5720 *m/z*; calcd. for $C_{63}H_{77}NNaO_9Si$ ($[M+Na]^+$): 1042.5260, found 1042.5262 *m/z*.

Compound 18

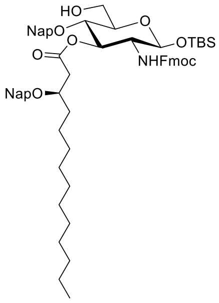

A mixture of compound **17** (1.5 g, 1.47 mmol) and molecular sieves (4 Å, 1.5 g) in extra dry CH_2Cl_2 (60 mL) was placed under an N_2 atmosphere and cooled to −78 °C. Triethylsilane (0.87 mL, 5.88 mmol) and $PhBCl_2$ (0.67 mL, 5.1 mmol) were then added. The reaction mixture was stirred at −78 °C for 1 h after which the reaction was quenched by the addition of methanol (6 mL) and triethylamine to a pH value of 8. Next, the mixture was filtered and washed with sat. aq. $NaHCO_3$ (10 mL). The organic layer was dried (anhydrous Na_2SO_4), filtered and concentrated in vacuo. The residue was purified by silica gel column chromatography (petroleum ether/ethyl acetate 6:1) to yield the acceptor compound **18** as colorless thick syrup (1.2 g, 80.1%). ^{1}H NMR (400 MHz, $CDCl_3$) δ 7.75 – 7.73 (1 H, m), 7.70 (7 H, dd, *J* = 14.7, 5.4), 7.66 (2 H, d, *J* = 5.3), 7.62 (2 H, d, *J* = 5.8), 7.53 (1 H, d, *J* = 3.9), 7.44 – 7.38 (6 H, m), 7.30 – 7.25 (3 H, m), 5.32 (1 H, t, *J* = 9.8,H-3), 4.93 (1 H, d, *J* = 9.3, NH), 4.79 (1 H, d, *J* = 6.6, H-1), 4.73 (2 H, q, *J* = 11.7, $NapCH_2$), 4.57 (2 H, dd, *J* = 24.5, 11.7, Fmoc-CH_2), 4.24 (2 H, d, *J* = 7.1, lipid-$NapCH_2$), 4.17 – 4.10 (1 H, m, Fmoc-CH), 3.85-3.80 (2 H, s, H-6, lipid-H-3), 3.74 (1 H, d, *J* = 3.9, H-4), 3.73 – 3.69 (1 H, m, H-6), 3.60 (1 H, dd, *J* = 18.4, 9.4, H-2), 3.49 (1 H, d, *J* = 8.3, H-5), 2.55 (1 H, dd, *J* = 15.6, 7.1), 2.39 (1 H, dd, *J* = 15.5, 4.9), 1.55 – 1.39 (2 H, m), 1.22 – 1.06 (18 H, m), 0.87 – 0.82 (12 H, m), 0.08 (6 H, d, *J* 13.4); ^{13}C NMR (101 MHz, $CDCl_3$) δ 172.00, 155.84, 143.86, 141.23, 135.99, 135.05, 133.23, 133.15, 132.98, 132.92, 128.26, 128.04, 127.95, 127.87, 127.69, 127.65, 127.04, 126.67, 126.29, 126.14, 126.00, 125.97, 125.87, 125.79, 125.74, 125.22, 119.96, 96.66 (H-1), 75.72 (H-4), 75.28 (H-5), 74.64 (H-3), 71.47 (Fmoc-CH_2), 67.24 (lipid-$NapCH_2$), 62.00 (H-6), 58.48 (H-2), 53.46, 47.06 (Fmoc-CH), 39.74, 34.19, 31.96, 29.67, 29.65, 29.62, 29.60, 29.38, 25.52, 25.15, 22.73, 17.90, 14.17, -4.07, -5.25. HRESI-MS calcd. for $C_{63}H_{79}NNaO_9Si$ ($[M+Na]^+$): 1044.5416, found 1044.5418 *m/z.*

Compound 19

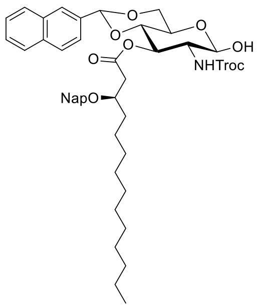

Compound **15** (1.6 g, 1.64 mmol) was dissolved in THF (48 mL), the solution cooled to −40 °C and HF/pyridine (4.8 mL, 65–70 %) in pyridine (30 mL) was added. Then, the reaction was allowed to warm to room temperature and stirred overnight. The reaction mixture was quenched by adding sat. aq. $NaHCO_3$ (80 mL) and diluted with CH_2Cl_2 (100 mL). The organic layer was dried (anhydrous Na_2SO_4), filtered, and the filtrate concentrated in vacuo. The residue was purified by silica gel column chromatography (petroleum ether/ethyl acetate 4:1) to give compound **19** as white solid (1.32 g, 93%). The product was confirmed by Q-TOF microMS analysis.

Compound 20

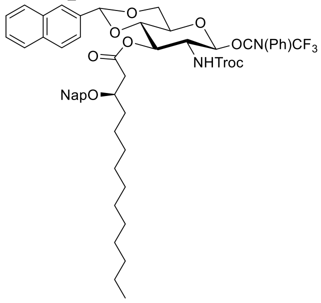

To a solution of compound **19** (0.65 g, 0.75 mmol) in dry CH_2Cl_2 (10 mL), 2,2,2-trifluoro-*N*-phenylacetimidoyl chloride (0.76 g, 4.5 mmol) and DBU (345 mg, 2.27 mmol) were added, and the reaction mixture was stirred for 30 min at room temperature. After complete consumption of the starting material as shown by TLC, the reaction mixture was concentrated and purified by silica gel column chromatography (petroleum ether/ethyl acetate 15:1 with 0.1% triethylamine) to afford donor **20** as colorless thick syrup (0.74 g, 95%). The product was confirmed by Q-TOF micro MS analysis.

Compound 21

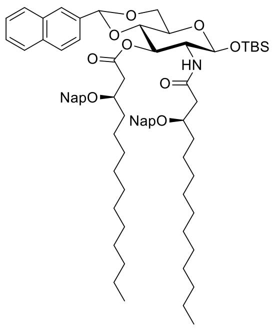

A solution of compound **15** (1.7 g, 1.75 mmol) in CH_2Cl_2 (40 mL) was placed under N_2 atmosphere, and Zn dust (3 g, 45.9 mmol) and acetic acid (4 mL) were added. After stirring at room temperature for 3 h, the reaction mixture was filtered and washed with sat. aq. $NaHCO_3$ (20 mL). The organic phase was dried (anhydrous Na_2SO_4) and concentrated. The deprotection of the Troc group was confirmed by Q-TOF microMS analysis. The crude free amine **16** (1.69 g, 2.12 mmol) was dissolved in CH_2Cl_2 (20 mL). Then, EDC·HCl (838 mg, 4.375 mmol), (*R*)-3-naphthyloxytetradecanoic acid **7** (1 g, 2.625 mmol), and DMAP (10 mg, 0.087 mmol) were added at room temperature and the reaction mixture was allowed to stir overnight. Upon completion, the reaction mixture was diluted with CH_2Cl_2 (20 mL) and washed with sat. aq. NaCl (20 mL). The organic phase was dried (anhydrous Na_2SO_4) and concentrated. The residue was purified by silica gel column chromatography (petroleum ether/ethyl acetate 10:1) to yield **21** as colorless thick syrup (1.37 g, 67.5% over two steps). ^{1}H NMR (400 MHz, $CDCl_3$) δ 7.98 – 7.51 (1 H, m), 6.47 (1 H, d, *J* = 9.3, N-H'), 5.47 (1 H, s), 5.28 (1 H, t, *J* = 9.9, H-3'), 4.90 – 4.47 (1 H, m), 4.35 (1 H, d, *J* = 7.8, H-1'), 4.23 (1 H, dd, *J* = 10.4, 4.9, H-6'), 3.97 (1 H, dd, *J* = 18.1, 9.5,H-2'), 3.91 – 3.80 (1 H, m), 3.74 (1 H, dd, *J* = 13.0, 7.5, H-6'), 3.70 – 3.61 (1 H, m, H-4'), 3.15 (1 H, tt, *J* = 15.9, 7.9, H-5'), 2.73 (1 H, dd, *J* = 14.7, 6.4), 2.54 – 2.41 (1 H, m), 1.69 – 1.48 (1 H, m), 1.28 (2 H, s), 0.94 – 0.88 (1 H, m), 0.07 – 0.01 (1 H, m); ^{13}C NMR (101 MHz, $CDCl_3$) δ 171.07, 170.59, 135.63, 135.35, 133.98, 133.22, 132.96, 132.80, 132.62, 132.49, 132.39, 128.05, 127.90, 127.60, 127.51, 127.34, 127.23, 126.17, 126.10, 125.91, 125.77, 125.62, 125.50, 125.29, 125.27, 123.27, 101.12, 96.63 (C-1'), 78.64 (C-4'), 75.58, 75.14, 71.11 (C-3'), 70.72, 70.31, 68.26 (C-6'), 65.74 (C-5'), 56.00 (C-2'), 40.64, 39.49, 34.25, 33.17, 31.56, 29.32, 29.29, 29.27, 29.20, 29.18, 28.99, 25.20, 24.86, 24.79, 22.32, 17.40, 13.77, -4.44, -5.60. HRESI-MS calcd. for $C_{73}H_{102}NO_9Si$ ($[M+H]^+$): 1164.7318, found 1164.7328 *m/z*; calcd. for $C_{73}H_{101}NNaO_9Si$ ($[M+Na]^+$): 1186.7138, found 1186.7110 *m/z*.

Compound 22

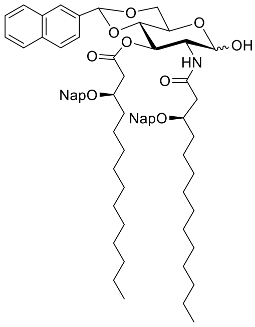

To a solution of compound **21** (1.2 g, 1.03 mmol) in tetrahydrofuran (6 mL), were added pyridine (18 mL) and a solution of HF/pyridine (3.6 mL, 65–70%) at −40 °C. Then, the reaction mixture was allowed to warm to room temperature and stirred overnight. The reaction mixture was then quenched by adding sat. aq. $NaHCO_3$ (20 mL) and diluted with CH_2Cl_2 (20 mL). The organic layer was dried (anhydrous Na_2SO_4), filtered, and the filtrate was concentrated in vacuo. The residue was purified by silica gel column chromatography (petroleum ether/ethyl acetate 6:1) to give compound **22** as a white solid (0.85 g, 78%). ^{1}H NMR (400 MHz, $CDCl_3$) δ 7.90 – 7.31 (1 H, m), 6.42 (1 H, d, *J* = 9.3, N-H'), 5.49 (1 H, s), 5.45 (1 H, t, *J* = 10.2, H-3'), 5.09 (1 H, d, *J* = 3.5, H-5'), 4.73 – 4.47 (1 H, m), 4.39 – 4.32 (1 H, m, H-4'), 4.22 (1 H, dd, *J* = 10.3, 4.9, H-1'), 4.08 (1 H, td, *J* = 9.9, 4.8, H-6'), 3.91 – 3.79 (1 H, m), 3.74 – 3.64 (1 H, m, H-2', H-6'), 2.74 – 2.67 (1 H, m), 2.51 – 2.43 (1 H, m), 2.32 (1 H, dd, *J* = 7.9, 3.4), 1.62 – 1.58 (1 H, m), 1.46 (1 H, ddd, *J* = 13.0, 10.3, 5.3), 1.22 (1 H, s), 0.88 (1 H, t, *J* = 6.5); ^{13}C NMR (101 MHz, $CDCl_3$) δ 174.90, 172.04, 171.43, 171.13, 135.49, 135.45, 135.24, 135.02, 133.81, 133.64, 133.17, 133.12, 132.84, 132.77, 132.70, 132.56, 132.44, 132.40, 132.29, 127.91, 127.82, 127.62, 127.56, 127.52, 127.48, 127.44, 127.40, 127.35, 127.25, 127.14, 127.12, 126.41, 125.87, 125.85, 125.81, 125.64, 125.53, 125.43, 125.42, 125.34, 125.19, 125.14, 123.18, 123.09, 101.23 (C-3'), 98.10 (C-1'), 91.92 (C-5'), 78.89 (C-2'), 78.02, 76.29, 75.00, 74.86, 73.43, 71.12, 69.26, 68.48 (C-6'), 68.11, 65.70, 62.31, 57.15, 52.23 (C-4'), 41.46, 41.02, 39.35, 39.21, 34.15, 33.95, 33.50, 33.44, 31.45, 29.21, 29.18, 29.17, 9.11, 28.89, 24.70, 22.22, 13.65, -0.48. HRESI-MS calcd. for $C_{67}H_{88}NO_9$ ($[M+H]^+$): 1050.6454, found 1050.6454 *m/z*; calcd. for $C_{67}H_{87}NNaO_9$ ($[M+Na]^+$): 1072.6273, found 1072.6257 *m/z*.

Compound 1

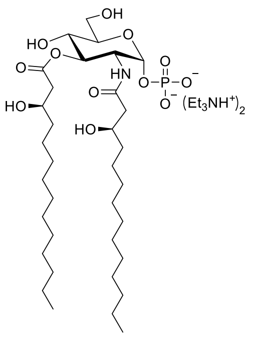

To a solution of compound **22** (0.15 g, 0.14 mmol) and tetrabenzyl pyrophosphate (385 mg, 0.71 mmol) in extra dry tetrahydrofuran (7.5 mL) was added LHMDS (0.63 mL) under N_2 atmosphere at −78 °C. The reaction mixture was stirred for 35 min. After complete consumption of the starting material as shown by TLC, the reaction was quenched by the addition of sat. aq. $NaHCO_3$ (20 mL) and diluted with CH_2Cl_2 (20 mL). The organic layer was dried (anhydrous Na_2SO_4), filtered, and the filtrate was concentrated in vacuo. The residue was purified by silica gel column chromatography (petroleum ether/ethyl acetate 12:1 with 0.1% TEA) to give compound **23** as white solid (0.17 g, 91%). HRESI-MS calcd. for $C_{81}H_{100}NNaO_{12}P$ ($[M+Na]^+$): 1332.6875, found 1332.6832 *m/z*.

Compound **23** (0.1 g, 0.076 mmol) was redissolved in THF/H_2O 85:15 (22 mL) and Pd/C (150 mg) was added. The reaction mixture was stirred under H_2 (15 kg/cm^2) at 38 °C for 12 h. The reaction mixture was quenched by adding one drop of triethylamine and filtered. The filtrate was concentrated in vacuo and purified by silica gel column chromatography (CH_2Cl_2/MeOH 5:1 with 0.1% TEA) to give compound **1** as white solid (46 mg, 86 %). ^{1}H NMR (400 MHz, $CDCl_3$) δ 5.27 (1 H, dd, *J* = 9.5, H-3'), 6.45 (1 H, d, *J* = 9.3), 5.25 (1 H, dd, *J*= 3.5,7.5, H-1'), 4.22 (1 H, ddd, *J* = 10.5, 2.5, H-2'), 4.00 (1 H, ddd, *J* = 5.6, H-5'), 3.90 (1 H, dd, *J* = 2.0, 1.2, H-6'), 3.87 (1 H, m), 3.82 (1 H, m), 3.75 (1 H, dd, *J* = 12, H-6'), 3.63 (1 H, dd, *J* = 10, H-4'), 2.75 – 2.67 (1 H, m), 2.51 – 2.42 (1 H, m), 2.32 (2 H, dd, *J* = 7.9, 3.4), 1.59 (1 H, dd, *J* = 14.5, 5.9), 1.47 (3 H, ddd, *J* = 14.5, 10.7, 5.2), 1.22 (36 H, s), 0.87 (6 H, d, *J* 7.1); ^{13}C NMR (151 MHz, $CDCl_3$) δ 174.54, 173.34, 95.10, 75.10, 74.27, 69.54, 69.11, 62.36, 53.32, 44.67, 43.41, 38.40, 38.04, 32.94, 30.66, 26.59, 23.60, 14.40. HRESI-MS calcd. for $C_{34}H_{65}NO_{12}P$ ($[M-H]^-$): 710.4250, found 710.4245 *m/z*. Ref. [4-5].

Compound 24

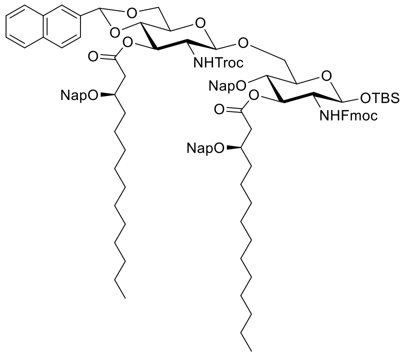

A mixture of glycosyl acceptor **18** (397 mg, 0.388 mmol), trifluorophenylacetimidoyl donor **20** (520 mg, 0.505 mmol) and molecular sieves (4 Å, 400 mg) in dry CH_2Cl_2 (2 mL) was placed under N_2 and cooled to −20 °C. Then, TfOH (13 µL, 0.155 mmol, in 0.6 mL CH_2Cl_2) was added and the resulting reaction mixture was stirred for 1 h at −20 °C. The reaction was quenched by the addition of methanol (5 mL) and triethylamine to a pH value of 8. Next, the mixture was diluted with CH_2Cl_2 (20 mL) and washed with sat. aq. $NaHCO_3$ (20 mL). The organic layer was dried (anhydrous Na_2SO_4), filtered, and the filtrate was concentrated in vacuo. The residue was purified by silica gel column chromatography (petroleum ether/ethyl acetate 12:1) to yield the disaccharide **24** as colorless syrup (681 mg, 94%). ^{1}H NMR (600 MHz, $CDCl_3$) δ 7.74 – 7.17 (36 H, m), 5.37 (1 H, s), 5.25 (2 H, dd, *J* = 21.8, 11.1, H-3, H-3’), 5.18 (1 H, t, *J* = 4.1, NH’), 4.88 (1 H, d, *J* = 9.1, NH), 4.68 (1 H, d, *J* = 7.7, H-1’), 4.67 – 4.40 (9 H, m, Fmoc-CH_2, NapCH_2, H-1), 4.21 (1 H, dd, *J* = 10.1, 4.6, H-6’), 4.17 (2 H, d, *J* = 7.1, H-6), 4.06 (1 H, d, *J* = 7.0), 3.83 (1 H, d, *J* = 10.8, H-6’), 3.76 (2 H, dd, *J* = 11.4, 5.7), 3.66 – 3.49 (6 H, m, H-2’, Troc, H-2, H-5, H-5’), 3.46 (1 H, s, H-4’), 3.31 (1 H, d, *J* = 4.4, H-4), 2.46 (4 H, dddd, *J* = 64.8, 44.5, 15.3, 5.6), 1.51 – 1.32 (4 H, m), 1.25 – 0.98 (36 H, m), 0.80 (9 H, t, *J* = 7.2), 0.78 (6 H, s); ^{13}C NMR (151 MHz, $CDCl_3$) δ 170.93, 170.43, 154.79, 153.10, 142.86, 142.80, 140.19, 134.94, 134.85, 134.31, 133.11, 132.56, 132.16, 132.13, 132.10, 131.72, 127.27, 127.18, 127.00, 126.97, 126.96, 126.83, 126.81, 126.67, 126.63, 126.62, 126.58, 126.00, 125.35, 125.28, 125.19, 125.06, 124.94, 124.90, 124.80, 124.70, 124.66, 124.57, 124.18, 122.54, 118.90, 100.46, 100.31 (C-1), 95.46 (C-1’), 94.42, 77.77, 74.92, 74.65, 74.42, 73.63, 73.39 (C-4),3.36 (C-3),70.44 (C-3’), 70.17, 70.12, 67.51 (C-6), 67.01, 66.16 (C-6’), 65.28 (C-4’), 57.23 (C-5), 55.74 (C-5’), 46.00, 38.71, 38.57, 33.49, 33.10, 30.89, 28.67, 28.59, 28.57, 28.51, 28.32, 24.55, 24.18, 24.07, 21.67, 16.84, 13.12, -4.94, -6.26.

Compound 26

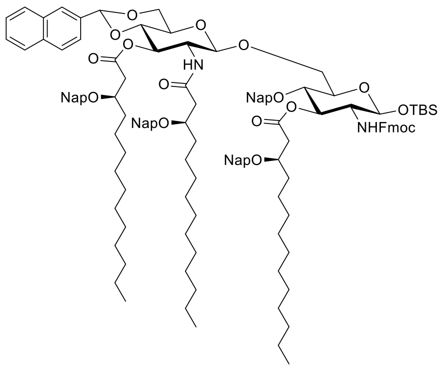

A solution of disaccharide **24** (267 mg, 0.14 mmol) in dry CH_2Cl_2 (3 mL) was placed under N_2 atmosphere, and Zn dust (0.9 g, 13.8 mmol) and acetic acid (0.9 mL) were added. After stirring at room temperature for 2 h, the reaction mixture was diluted with $CHCl_3$ (20 mL) and filtered through Celite. The filtrate was concentrated and the residue was co-evaporated three times with toluene. The removal of the Troc protecting group gave compound **25** that was purified by silica gel column chromatography (petroleum ether/ethyl acetate 5:1). The crude free amine **25** (180 mg) was redissolved in dry CH_2Cl_2 (10 mL) followed by the addition of EDC·HCl (246 mg, 1.28 mmol) and (*R*)-3-naphthyloxytetradecanoic acid **7** (165 mg, 0.43 mmol) in dry CH_2Cl_2 (5 mL) at –10°C under N_2 atmosphere. The reaction mixture was stirred for 12 h. After complete consumption of the starting material as shown by TLC, the reaction mixture was concentrated and purified by silica gel column chromatography (petroleum ether/ethyl acetate = 10:1) to give compound **26** as a light yellow solid (0.223 g, 88%). ^{1}H NMR (600 MHz, $CDCl_3$) δ 7.80 – 7.15 (43 H, m), 6.31 (1 H, d, *J* = 8.9, NH’), 5.32 (1 H, s), 5.18 (1 H, t, *J* = 9.8, H-3’), 5.12 (1 H, t, *J* = 9.7, H-3), 4.75 (1 H, d, *J* = 9.2, NH), 4.64 – 4.38 (11 H, m, H-1’, $NapCH_2$, Fmoc), 4.16 (2 H, d, *J* = 7.3, H-6’), 4.14 – 4.09 (2 H, m, H-1, H-6), 4.08 – 4.04 (1 H, m, FmocCH), 3.99 – 3.93 (1 H, m, H-2’), 3.74 (3 H, dt, *J* = 17.7, 8.8, lipid-H-3*2, H-6’), 3.66 – 3.48 (5 H, m, H-2, H-6’, lipid-H-3, H-5, H-5’), 3.35 (1 H, s, H-4’), 3.07 (1 H, dd, *J* = 14.5, 9.6, H-4), 2.64 – 2.20 (6 H, m), 1.44 – 1.29 (6 H, m), 1.24 – 1.10 (54 H, m), 0.80 (15 H, t, *J* = 7.1), 0.76 (9 H, s); ^{13}C NMR (151 MHz, $CDCl_3$) δ 170.85, 170.45, 170.28, 142.88, 142.84, 140.18, 134.98, 134.92, 134.35, 133.22, 132.58, 132.28, 132.16, 132.13, 131.89, 131.88, 131.84, 131.73, 127.35, 127.26, 127.11, 126.96, 126.89, 126.88, 126.85, 126.82, 126.73, 126.62, 126.58, 125.99, 125.43, 125.28, 125.21, 125.17, 125.14, 125.09, 124.99, 124.88, 124.80, 124.72, 124.65, 124.19, 122.59, 118.89, 100.46, 100.41 (C-1), 95.46 (C-1’),77.74 (C-5), 75.26, 75.17 (C-5’), 74.87, 74.59, 74.46, 73.52 (C-4’), 73.33 (C-3), 70.74 (C-3’), 70.61, 70.39, 70.08, 69.89, 67.54 (C-6), 66.84 (C-6’), 66.10, 65.20 (C-4), 57.15, 53.14 (C-2), 46.01 (C-2’), 40.29, 38.78, 38.64, 38.09, 33.58, 33.18, 32.74, 30.90, 28.67, 28.56, 28.35, 28.33, 24.42, 24.09, 23.95, 21.67, 16.83, 13.11, -4.88, -6.31. HRMS (MALDI) calcd. for $C_{130}H_{164}N_2KO_{17}Si$ ($[M+K]^+$): 2092.1431, found 2092.0216 *m/z*.

Compound 28

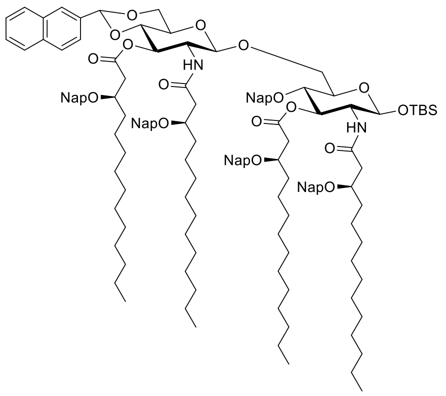

To a solution of compound **26** (216 mg, 0.105 mmol) in DMF (4 mL) was added triethylamine (4 mL) under N_2. After stirring at room temperature for 12 h, the reaction mixture was concentrated and purified by silica gel column chromatography (petroleum ether/ethyl acetate 6:1). The crude free amine **27** was redissolved in dry CH_2Cl_2 (3 mL) followed by the addition of EDC·HCl (320 mg, 1.67 mmol) and (*R*)-3-naphthyloxytetradecanoic acid (**7**, 320 mg, 0.83 mmol) at room temperature. The reaction mixture was stirred for 12 h. After complete consumption of the starting material as shown by TLC, the reaction mixture was concentrated and purified by silica gel column chromatography (petroleum ether/ethyl acetate 12:1) to give compound **28** as light yellow solid (189 mg, 82%). ^{1}H NMR (600 MHz, $CDCl_3$) δ 7.83 – 7.13 (42 H, m), 6.31 (1 H, d, *J* = 9.0, NH'), 6.14 (1 H, d, *J* = 9.3, NH), 5.28 (1 H, d, *J* = 4.5), 5.15 (1 H, t, *J* = 9.8, H-3'), 5.07 – 5.03 (1 H, t, H-3), 4.66 – 4.38 (10 H, m), 4.24 (1 H, d, *J* = 7.7, H-1), 4.06 (1 H, dd, *J* = 10.4, 4.9, H-6'), 4.03 (1 H, d, *J* = 8.3, H-1'), 3.90 (1 H, dd, *J* = 18.6, 9.3, H-2'), 3.79 – 3.71 (3 H, m, H-2, lipid-H-3*2), 3.70 – 3.68 (1 H, m, lipid-H-3), 3.64 (1 H, d, *J* = 9.8, H-6'), 3.59 (1 H, s, lipid-H-3), 3.50 (2 H, dd, *J* = 14.2, 6.9, H-5',H-6), 3.46 (1 H, t, *J* = 9.5, H-5), 3.15 (1 H, s, H-4), 3.00 (1 H, td, *J* = 9.7, 5.2, H-4'), 2.33 (16 H, ddddd, *J* = 52.4, 24.7, 21.3, 15.1, 7.1), 1.27 – 1.04 (72 H, m), 0.80 (21 H, dd, *J* = 9.2, 4.9), 0.75 (9 H, s); ^{13}C NMR (151 MHz, $CDCl_3$) δ 170.57, 170.41, 170.22, 169.89, 135.06, 134.93, 134.83, 134.77, 134.47, 133.24, 132.57, 132.30, 132.29, 132.17, 132.15, 132.13, 131.94, 131.90, 131.84, 131.73, 128.89, 128.86, 127.35, 127.26, 127.04, 126.95, 126.87, 126.83, 126.70, 126.67, 126.61, 126.58, 125.42, 125.37, 125.28, 125.26, 125.21, 125.14, 125.12, 125.10, 125.08, 124.98, 124.92, 124.87, 124.82, 124.80, 124.73, 124.64, 124.62, 124.54, 123.44, 122.96, 122.60, 118.07, 100.42, 100.32 (C-1), 95.22 (C-1'), 77.73 (C-5), 75.24, 74.99, 74.83, 74.55, 74.46 (C-3), 73.52 (C-5'), 73.11 (C-4), 73.06, 70.71 (C-3'), 70.32, 70.08, 69.85, 69.65, 67.52 (C-6'), 66.89 (C-6), 65.11 (C-4'), 54.86 (C-2), 53.15 (C-2'), 40.18, 40.15, 38.78, 38.70, 36.08, 33.85, 33.59, 33.50, 33.24, 32.69, 30.91, 30.61, 30.41, 29.17, 28.68, 28.64, 28.56, 28.35, 26.19, 24.67, 24.23, 24.14, 23.95, 21.68, 16.78, 13.11, -0.00, -4.78, -6.18. HRMS (MALDI) calcd. for $C_{140}H_{188}N_2NaO_{17}Si$ ($[M+Na]^+$): 2220.3570, found 2220.1805 *m/z*.

Compound 29

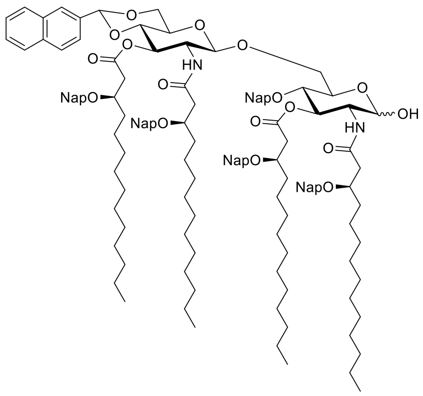

To a solution of compound **28** (548 mg, 0.25 mmol) in tetrahydrofuran (15 mL), pyridine (5.25 mL), and a solution of HF/pyridine (1.75 mL, 65–70%) were added at −40 °C. The reaction mixture was allowed to warm to room temperature and stirred overnight. The reaction mixture then was quenched by adding sat. aq. $NaHCO_3$ (30 mL) and diluted with CH_2Cl_2 (30 mL). The organic layer was dried (anhydrous Na_2SO_4), filtered, and the filtrate concentrated in vacuo. The residue was purified by silica gel column chromatography (petroleum ether/ethyl acetate 6:1) to give compound **29** as white solid (477 mg, 92%). ^1H NMR (600 MHz, $CDCl_3$) δ 7.87 – 7.14 (42 H, m), 6.46 (2 H, dd, *J* = 23.0, 9.0, NH, NH'), 5.42 (1 H, d, *J* = 9.4, H-1'), 5.28 (1 H, t, *J* = 8.9, H-3), 5.02 (1 H, d, *J* = 3.2, H-3'), 4.71 – 4.45 (8 H, m), 4.40 (1 H, d, *J* = 7.8, H-1), 4.21 (2 H, dd, *J* = 9.7, 5.5, H-2', H-6'), 3.96 – 3.80 (7 H, m, H-2, H-5', lipid-H-3, H-6), 3.74 (1 H, s, H-6'), 3.67 (1 H, t, *J* = 10.2, H-5), 3.60 (1 H, t, *J* = 9.4, H-4), 3.34 (2 H, t, *J* = 9.2, H-6, H-4'), 2.70 – 2.10 (12 H, m), 1.45 (4 H, ddd, *J* = 26.2, 23.9, 21.1), 1.23 (72 H, dt, *J* = 28.4, 18.1), 0.91 – 0.86 (12 H, m); ^{13}C NMR (151 MHz, $CDCl_3$) δ 170.97, 170.89, 170.39, 135.01, 134.99, 134.91, 134.06, 133.96, 133.17, 132.59, 132.49, 132.41, 132.29, 132.20, 132.17, 132.13, 132.08, 132.00, 131.93, 131.87, 131.84, 131.73, 129.09, 128.82, 128.74, 128.72, 127.53, 127.38, 127.36, 127.34, 127.26, 127.07, 127.03, 127.00, 126.92, 126.89, 126.88, 126.82, 126.69, 126.68, 126.59, 126.56, 125.88, 125.41, 125.36, 125.29, 125.23, 125.11, 125.08, 125.02, 125.00, 124.92, 124.91, 124.85, 124.67, 124.62, 122.58, 120.32, 101.67 (C-1)101.10 (C-1'), 100.56, 90.33 (C-3'), 78.36 (C-4'),74.56 (C-5'), 74.44, 73.12, 72.38, 71.88 (C-3), 70.78, 70.68, 70.52, 70.36, 70.17, 70.01, 69.63 (C-5'), 69.19, 68.79 (C-6), 68.38 (C-6'), 67.56 (C-4'), 65.30, 62.13, 54.49 (C-2), 53.93, 52.19 (C-2'), 40.93, 40.43, 38.85, 38.72, 34.39, 33.52, 33.28, 33.17, 32.71, 30.92, 30.41, 29.17, 28.68, 28.64, 28.62, 28.56, 28.36, 24.20, 24.17, 24.12, 24.03, 21.68, 15.23, 13.11. HRMS (MALDI) calcd. for $C_{134}H_{174}N_2NaO_{17}$ ($[M+Na]^+$): 2106.2705, found 2106.1674 *m/z*.

Compound 2

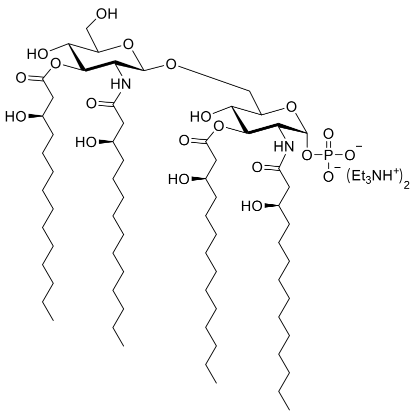

To a solution of compound **29** (200 mg, 0.1 mmol) and tetrabenzyl pyrophosphate (256 mg, 0.475 mmol) in extra dry tetrahydrofuran (8 mL) was added LHMDS (0.8 mL) under N_2 atmosphere at −78 °C. The reaction mixture was stirred for 1 h at −78 °C. After complete consumption of the starting material as shown by TLC, the reaction was quenched by the addition of methanol (5 mL), concentrated in vacuo and purified by silica gel column chromatography (petroleum ether/ethyl acetate 10:1 with 0.1% TEA) to give the crude product **30** as white solid (184 mg, 82%); Compound **30** (184 mg, 0.078 mmol) was redissolved in tetrahydrofuran (20 mL) and Pd black (1.4 g) was added. The reaction mixture was stirred under H_2 (15 kg/cm^2) at 38 °C for 24 h. The reaction was then quenched by one drop of triethylamine. The filtrate was concentrated in vacuo and the residue was purified by silica gel column chromatography (CH_2Cl_2/MeOH 5:1 with 0.1% TEA) to give compound **2** as white solid (91 mg, 88%). ^{1}H NMR (600 MHz, $CDCl_3$:CD_3OD = 2:1) δ 4.90 (1 H, t, *J* = 12.4), 4.31 (3 H, d, *J* = 7.6), 4.10 – 4.02 (2 H, m), 3.86 (2 H, d, *J* = 11.7), 3.78 – 3.70 (3 H, m), 3.67 (1 H, d, *J* = 6.0), 3.29 (4 H, dd, *J* = 16.5, 10.4), 3.13 (2 H, d, *J* = 6.8), 3.07 – 3.00 (2 H, m), 2.87 (2 H, s), 2.02 (4 H, s), 1.48 (8 H, dd, *J* = 13.6, 6.4), 1.32 – 1.22 (72 H, m), 0.89 (12 H, t, *J* = 6.0); ^{13}C NMR (151 MHz, $CDCl_3$:CD_3OD = 2:1) δ 171.4, 169.3, 165.0, 109.2, 98.0, 81.4, 80.1, 75.0, 74.5, 69.3, 67.8, 67.7, 68.4, 61.9, 57.4, 57.0, 42.3, 44.1, 36.8, 34.5, 31.9, 29.9, 29.6, 29.3, 25.1, 24.7, 22.7, 14.1. HRESI-MS calcd. for $C_{68}H_{128}N_2O_{20}P$ ([M-H]$^-$): 1323.8804, found 1324.8803 *m/z*. Ref. [6-7].

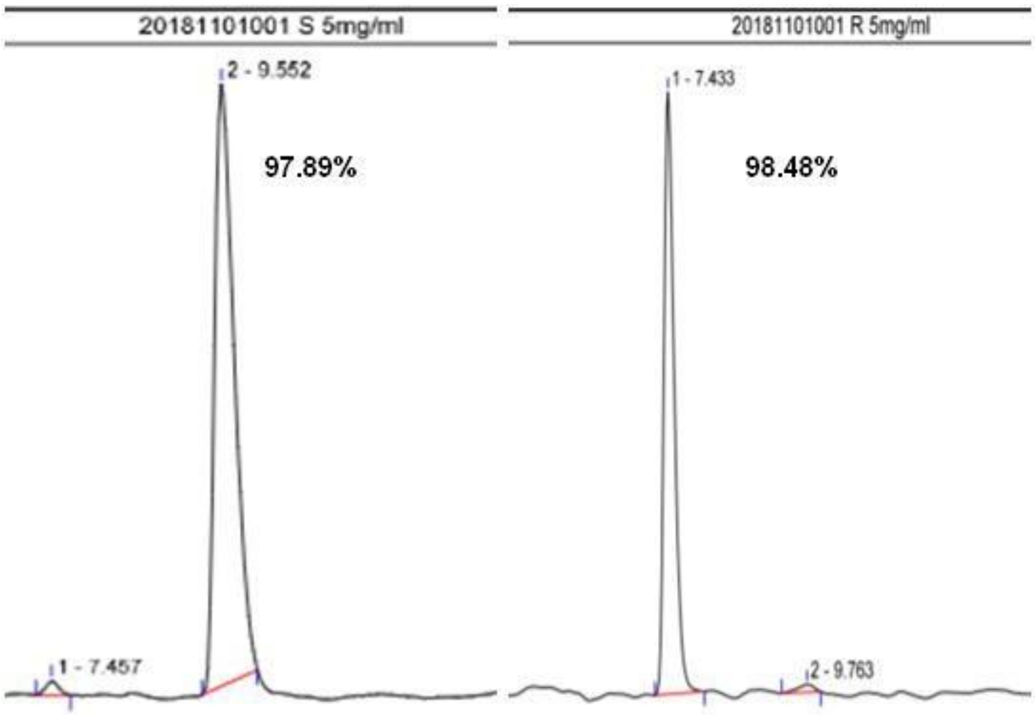

Figure S1: Chiral HPLC used to confirm the absolute configuration and enantiomeric purity of the 3-hydroxytetradecanoic acid methyl ester (**5**) from enantioselective hydrogenation.

References

(1) Kücük, H. B.; Yusufoğlu, A. *Monatshefte für Chemie - Chemical Monthly* **2013**, *144*, 1087-1091. doi: 10.1007/s00706-012-0917-z

(2) Boons, G.-J.; Wang, Z. Compounds and Methods for Chemical and Chemo-Enzymatic Synthesis of Complex Glycans. WIPO/PCT WO/2012/135049 A1, October 4, 2012.

(3) Mo, K.-F.; Fang, T.; Stalnaker, S. H.; Kirby, P. S.; Liu, M.; Wells, L.; Pierce, M.; Live, D. H.; Boons, G.-J. *J. Am. Chem. Soc.* **2011**, *133*, 14418-14430. doi: 10.1021/ja205473q

(4) Miyamoto, M.; Baker, M. L.; Lewis, M. D. *Tetrahedron Lett.* **1992**, *33*, 3725-3728. doi: 10.1016/0040-4039(92)80009-9

(5) Ribeiro, A. A.; Zhou, Z.; Raetz, C. R. H. *Magnetic Resonance in Chemistry* **1999**, *37*, 620-630. doi: 10.1002/(sici)1097-458x(199909)37:9<620::aid-mrc517>3.0.co;2-q

(6) Imoto, M.; Yoshimura, H.; Yamamoto, M.; Shimamoto, T.; Kusumoto, S.; Shiba, T. *Tetrahedron Lett.* **1984**, *25*, 2667-2670. doi: 10.1016/S0040-4039(01)81258-2

(7) Scholz, D.; Bednarik, K.; Ehn, G.; Neruda, W.; Janzek, E.; Loibner, H.; Briner, K.; Vasella, A. *J. Med. Chem.* **1992**, *35*, 2070-2074. doi: 10.1021/jm00089a019

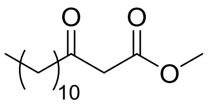
O
O
O
10

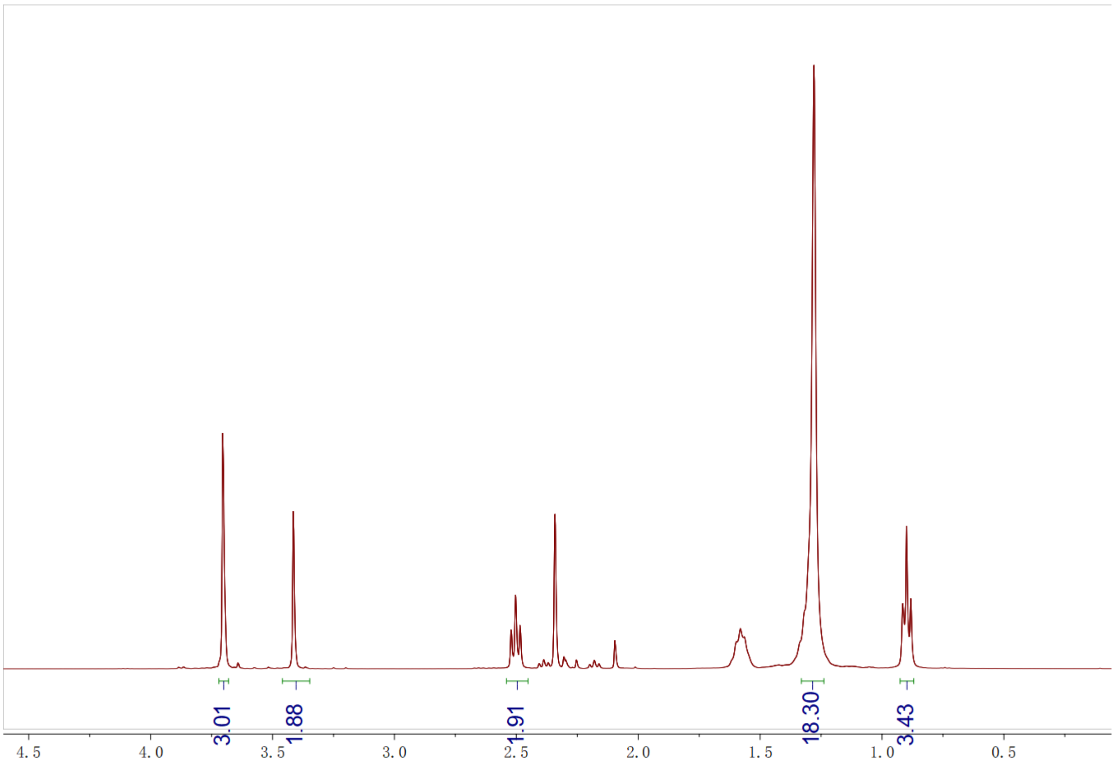
3.01
1.88
1.91
18.30
3.43
4.5
4.0
3.5
3.0
2.5
2.0
1.5
1.0
0.5

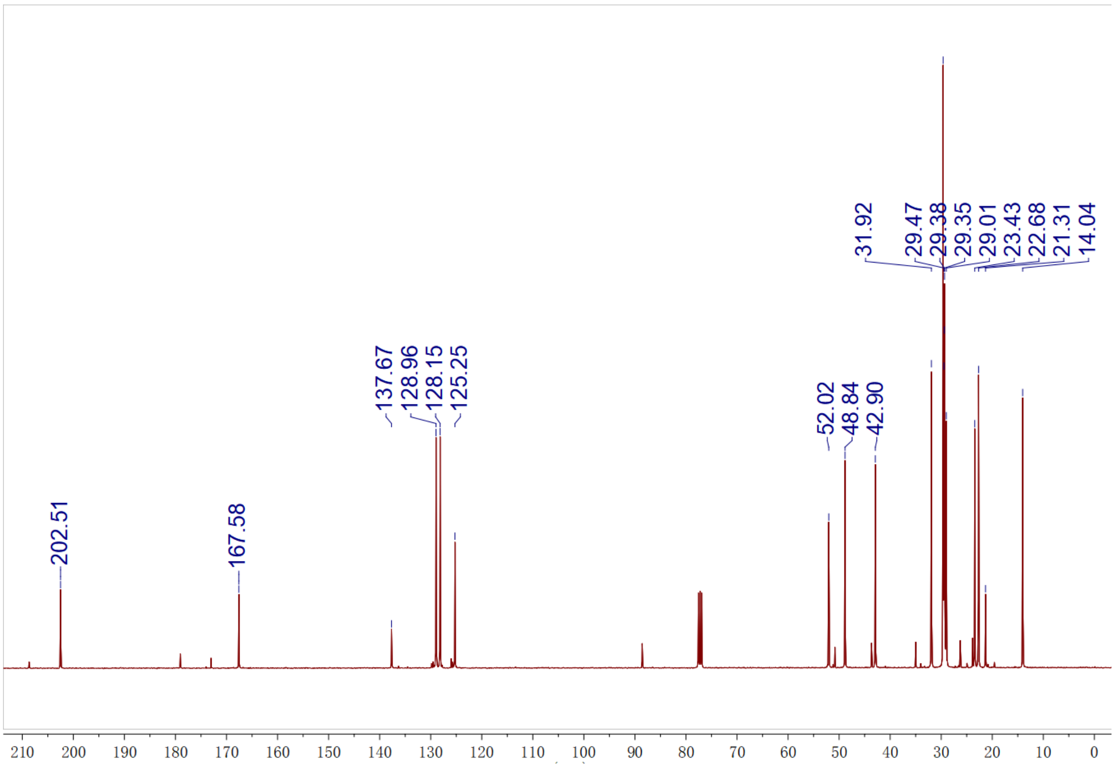
202.51
167.58
137.67
128.96
128.15
125.25
52.02
48.84
42.90
31.92
29.47
29.38
29.35
29.01
23.43
22.68
21.31
14.04
210
200
190
180
170
160
150
140
130
120
110
100
90
80
70
60
50
40
30
20
10
0

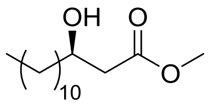

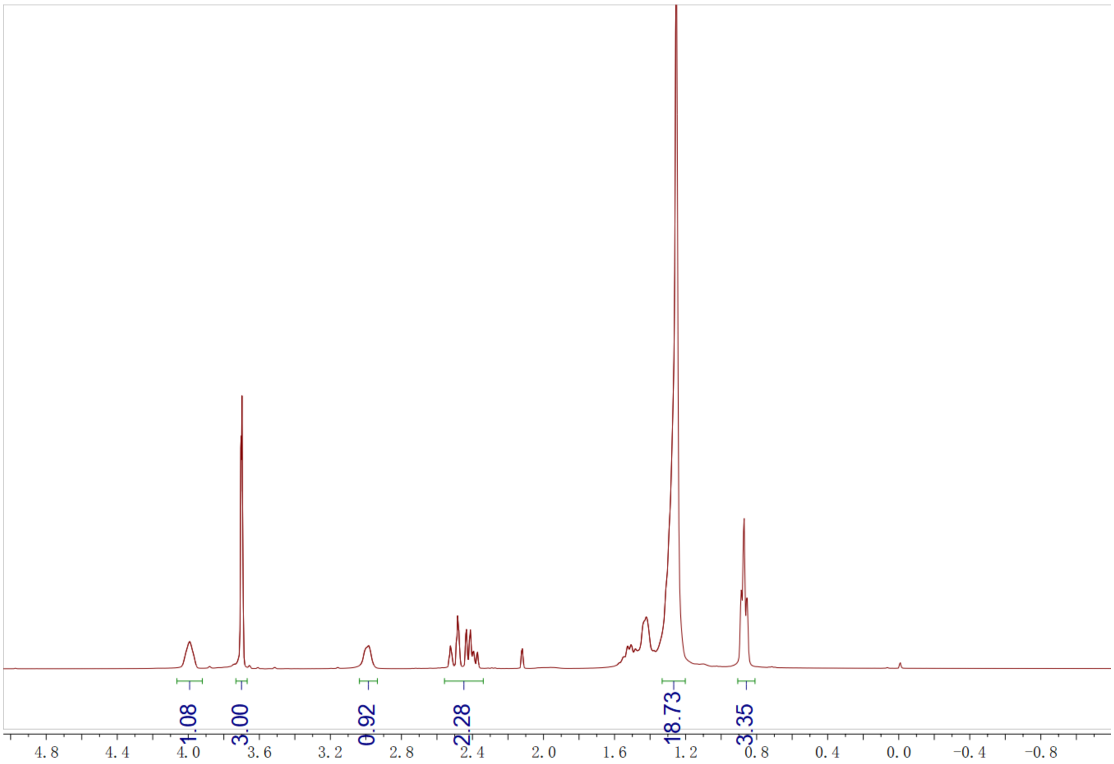
1.08
3.00
0.92
2.28
18.73
3.35
4.8
4.4
4.0
3.6
3.2
2.8
2.4
2.0
1.6
1.2
0.8
0.4
0.0
−0.4
−0.8

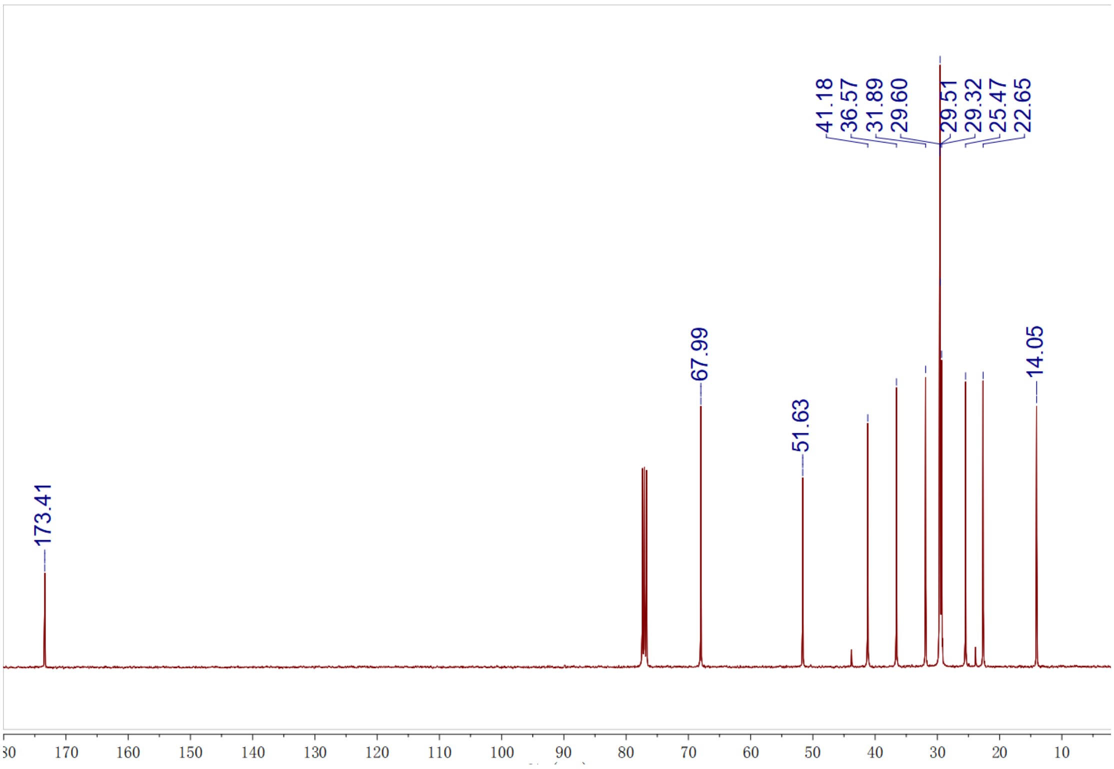
41.18
36.57
31.89
29.60
29.51
29.32
25.47
22.65
67.99
51.63
14.05
173.41
170
160
150
140
130
120
110
100
90
80
70
60
50
40
30
20
10

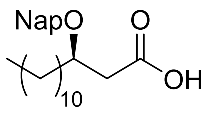

NapO
O
OH
10

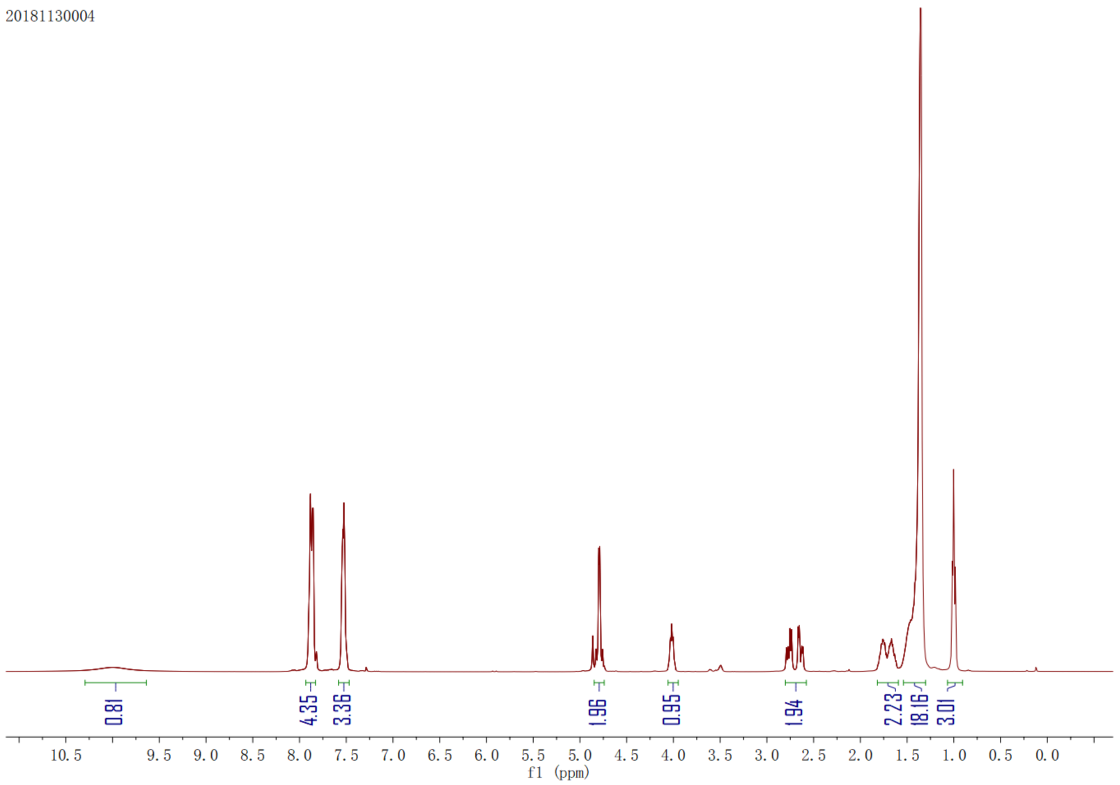

20181130004
0.81
4.35
3.36
1.96
0.95
1.94
2.23
18.16
3.01
10.5
9.5
9.0
8.5
8.0
7.5
7.0
6.5
6.0
5.5
5.0
4.5
4.0
3.5
3.0
2.5
2.0
1.5
1.0
0.5
0.0
f1 (ppm)

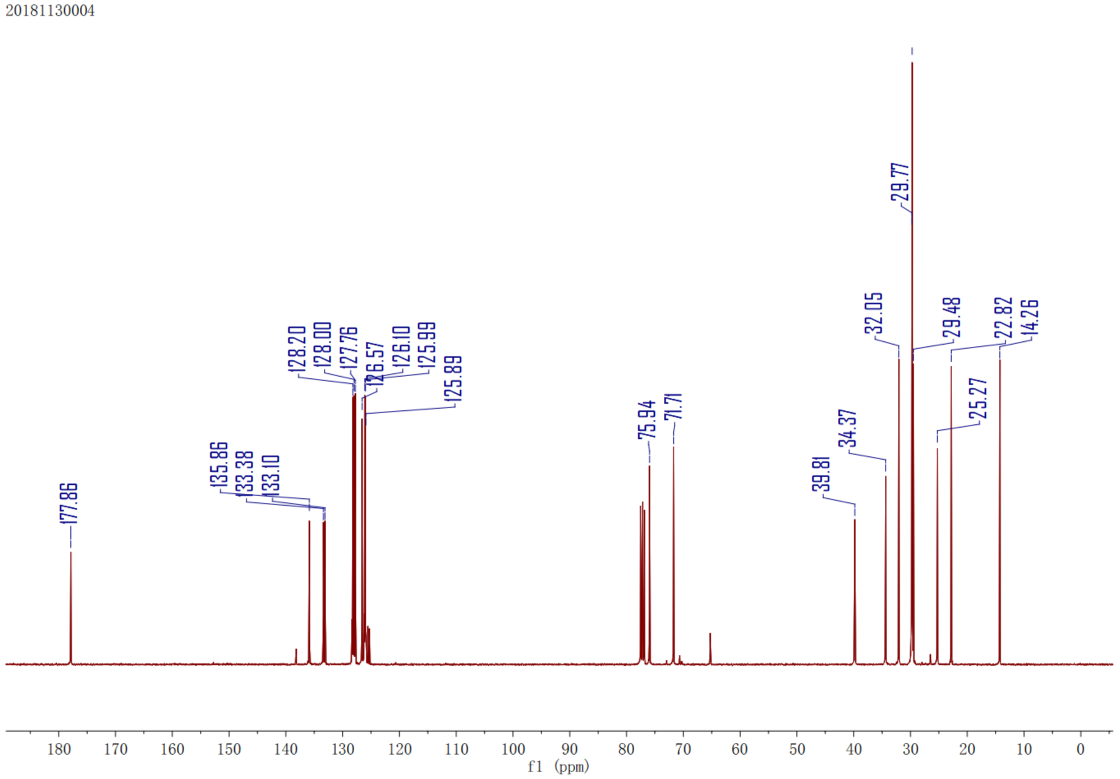

20181130004
177.86
135.86
133.38
133.10
128.20
128.00
127.76
126.57
126.10
125.99
125.89
75.94
71.71
39.81
34.37
32.05
29.77
29.48
25.27
22.82
14.26
180
170
160
150
140
130
120
110
100
90
80
70
60
50
40
30
20
10
0
f1 (ppm)

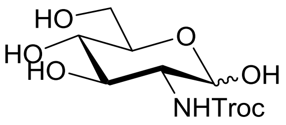
HO
HO
HO
O
OH
NHTroc

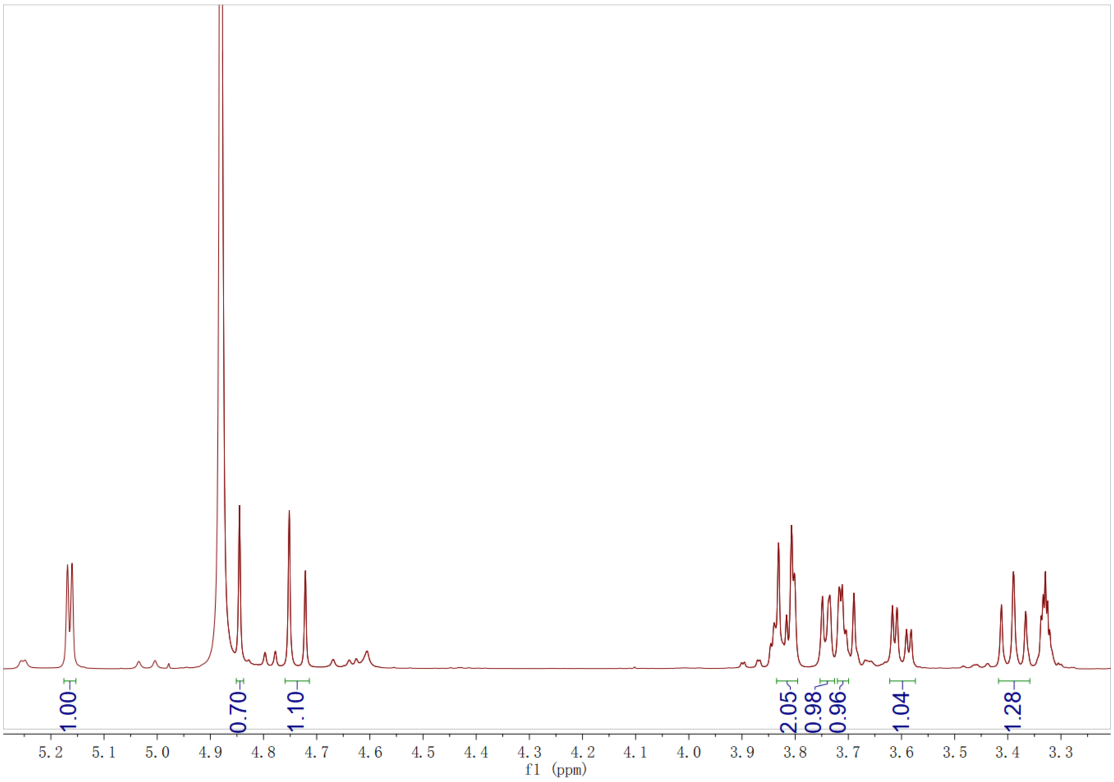
1.00
0.70
1.10
2.05
0.98
0.96
1.04
1.28
5.2 5.1 5.0 4.9 4.8 4.7 4.6 4.5 4.4 4.3 4.2 4.1 4.0 3.9 3.8 3.7 3.6 3.5 3.4 3.3
f1 (ppm)

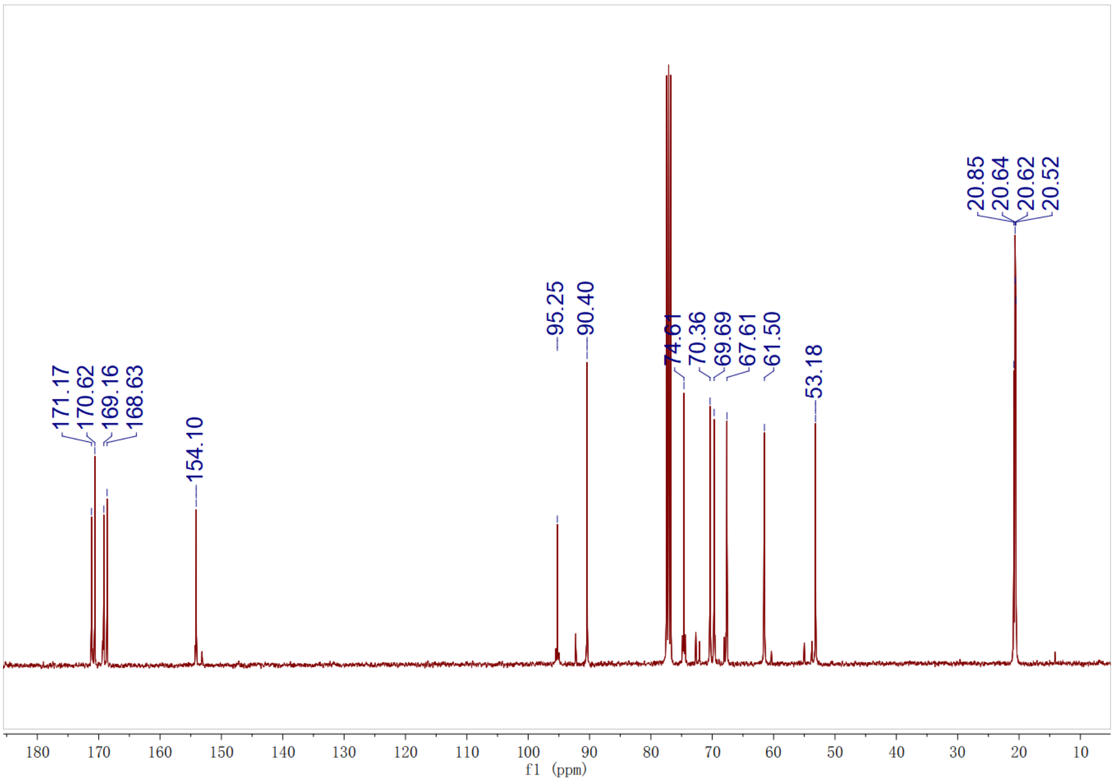
171.17
170.62
169.16
168.63
154.10
95.25
90.40
74.61
70.36
69.69
67.61
61.50
53.18
20.85
20.64
20.62
20.52
180 170 160 150 140 130 120 110 100 90 80 70 60 50 40 30 20 10
f1 (ppm)

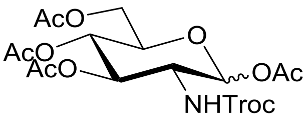
AcO
O
AcO
AcO
OAc
NHTroc

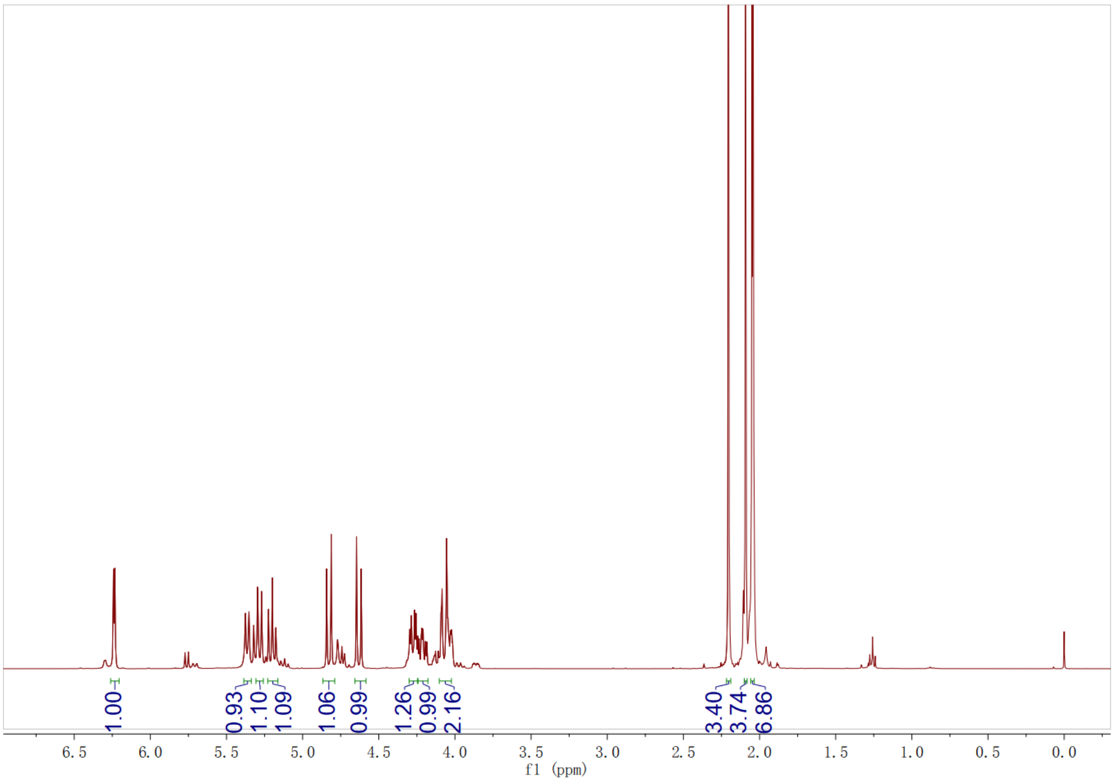
1.00
0.93
1.10
1.09
1.06
0.99
1.26
0.99
2.16
3.40
3.74
6.86
6.5
6.0
5.5
5.0
4.5
4.0
3.5
3.0
2.5
2.0
1.5
1.0
0.5
0.0
f1 (ppm)

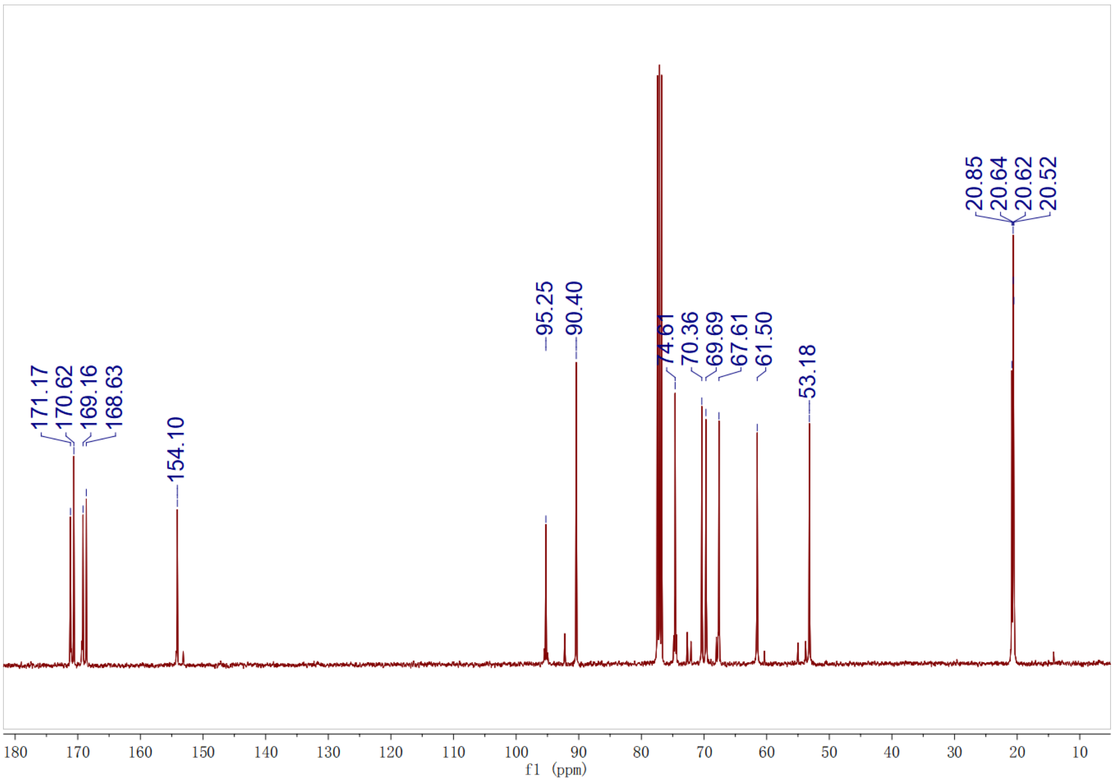
171.17
170.62
169.16
168.63
154.10
95.25
90.40
74.61
70.36
69.69
67.61
61.50
53.18
20.85
20.64
20.62
20.52
180
170
160
150
140
130
120
110
100
90
80
70
60
50
40
30
20
10
f1 (ppm)

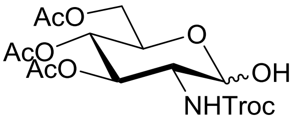
AcO
O
AcO
AcO
OH
NHTroc

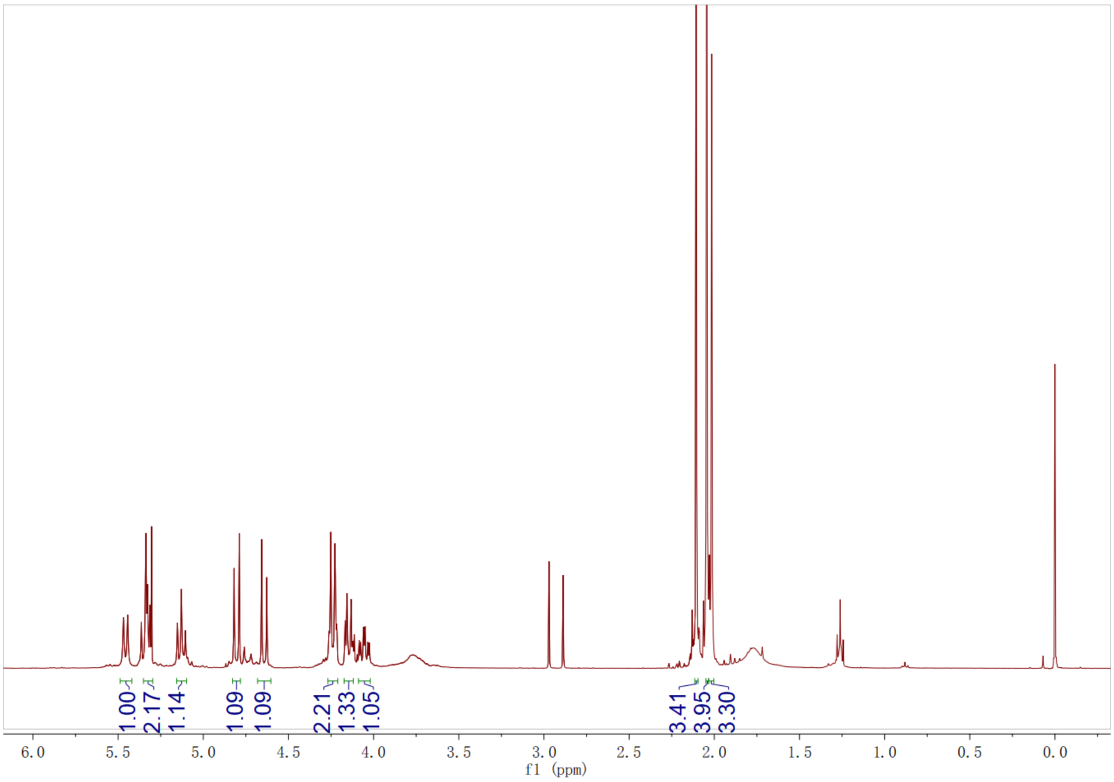
1.00
2.17
1.14
1.09
1.09
2.21
1.33
1.05
3.41
3.95
3.30
6.0
5.5
5.0
4.5
4.0
3.5
3.0
2.5
2.0
1.5
1.0
0.5
0.0
f1 (ppm)

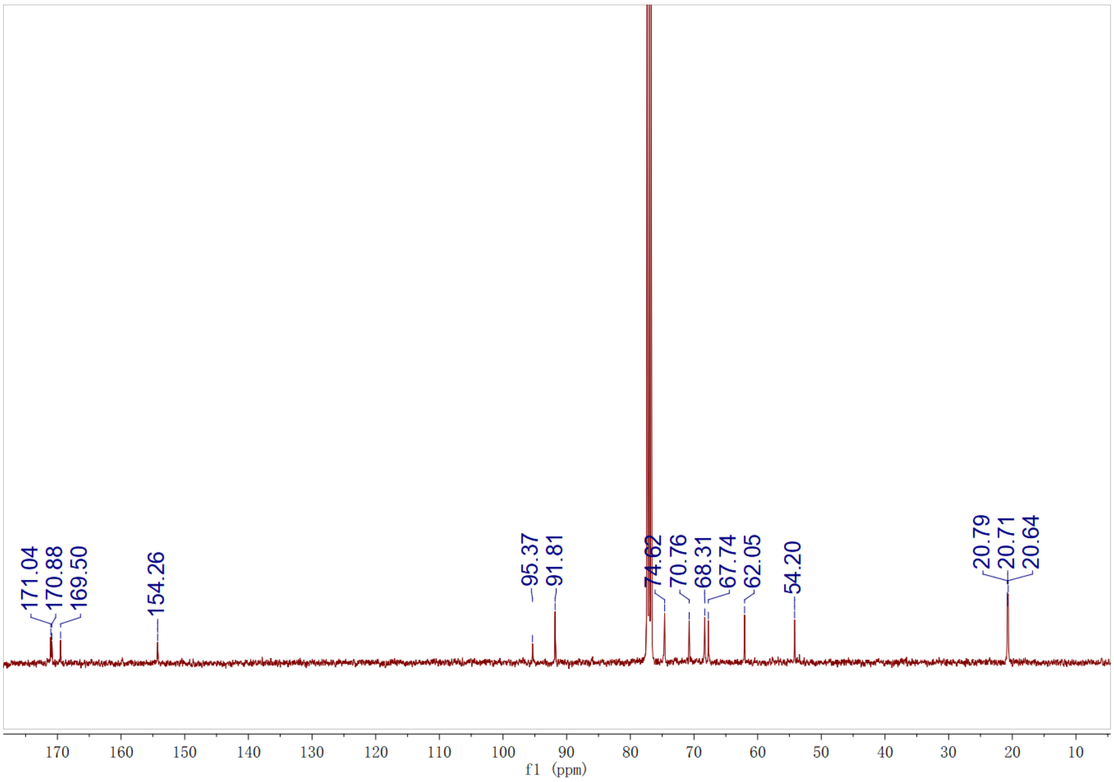
171.04
170.88
169.50
154.26
95.37
91.81
74.62
70.76
68.31
67.74
62.05
54.20
20.79
20.71
20.64
170
160
150
140
130
120
110
100
90
80
70
60
50
40
30
20
10
f1 (ppm)

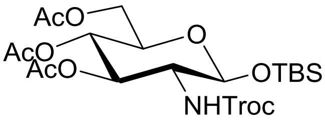

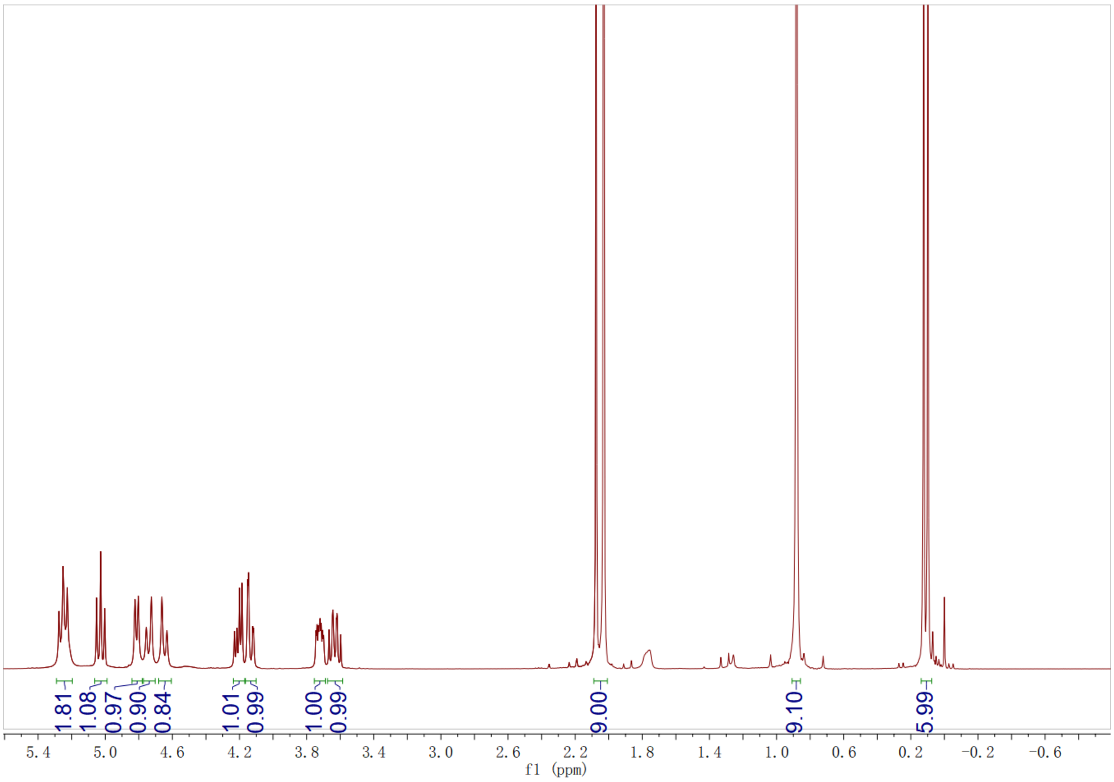
1.81
1.08
0.97
0.90
0.84
1.01
0.99
1.00
0.99
9.00
9.10
5.99
5.4
5.0
4.6
4.2
3.8
3.4
3.0
2.6
2.2
1.8
1.4
1.0
0.6
0.2
-0.2
-0.6
f1 (ppm)

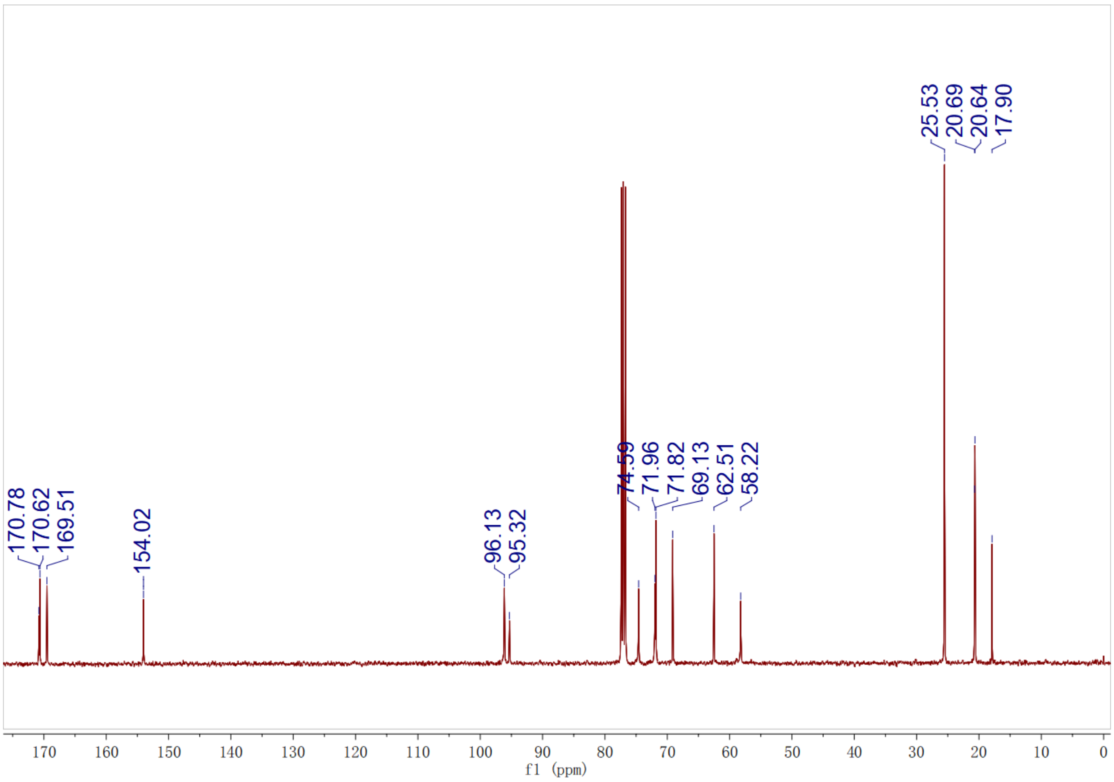
25.53
20.69
20.64
17.90
74.59
71.96
71.82
69.13
62.51
58.22
170.78
170.62
169.51
154.02
96.13
95.32
170
160
150
140
130
120
110
100
90
80
70
60
50
40
30
20
10
0
f1 (ppm)

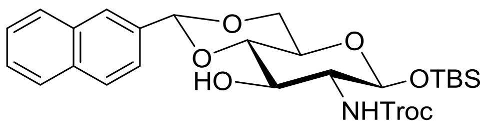

20181217034

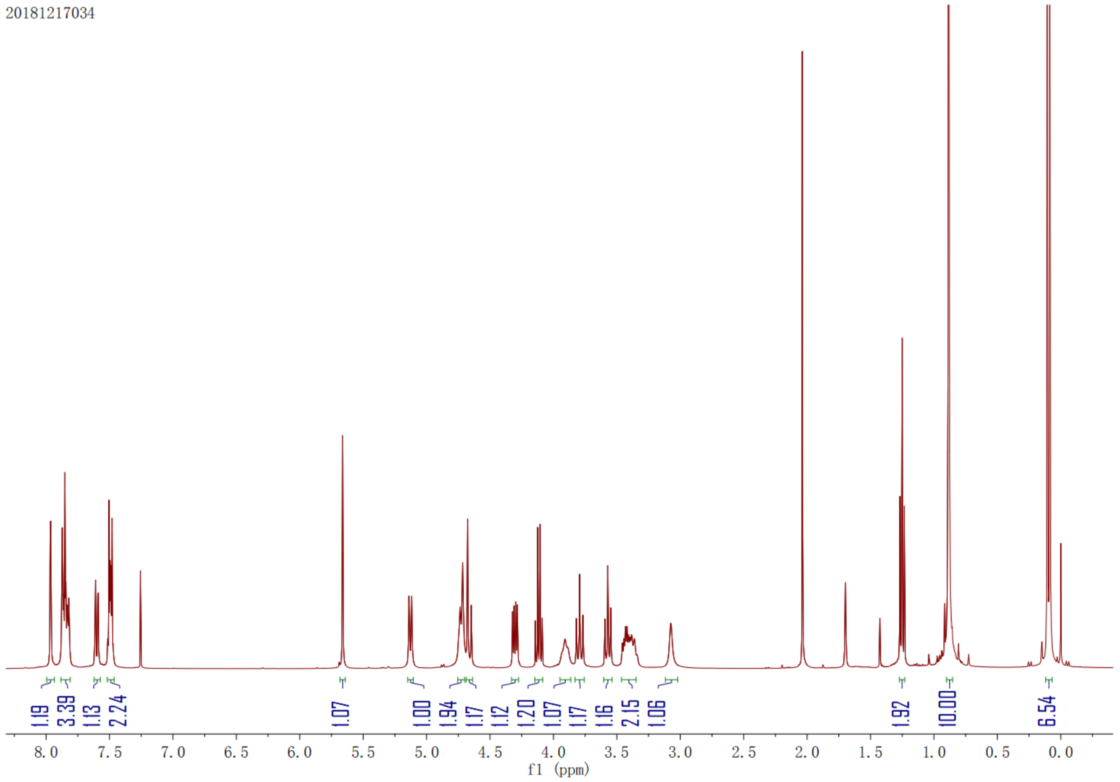

yigongyuan
20181010010-cdcl3-181016-13c

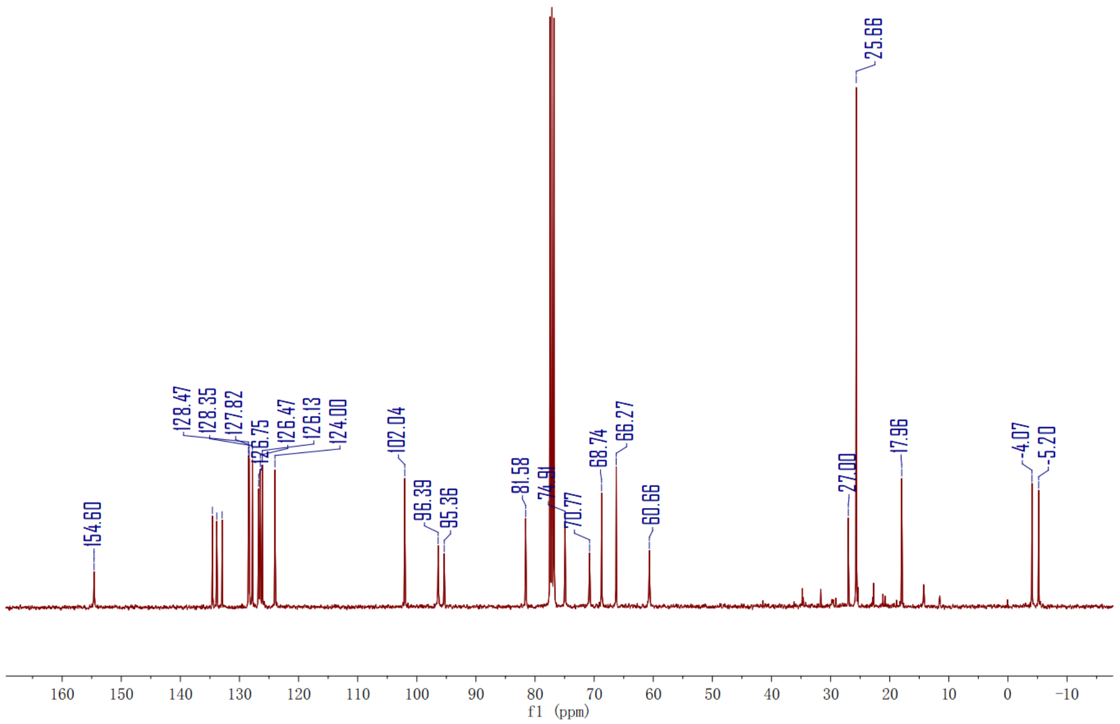

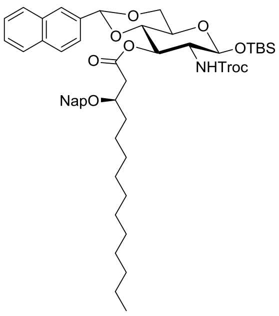

O
O
O
O
OTBS
O
NHTroc
O
NapO

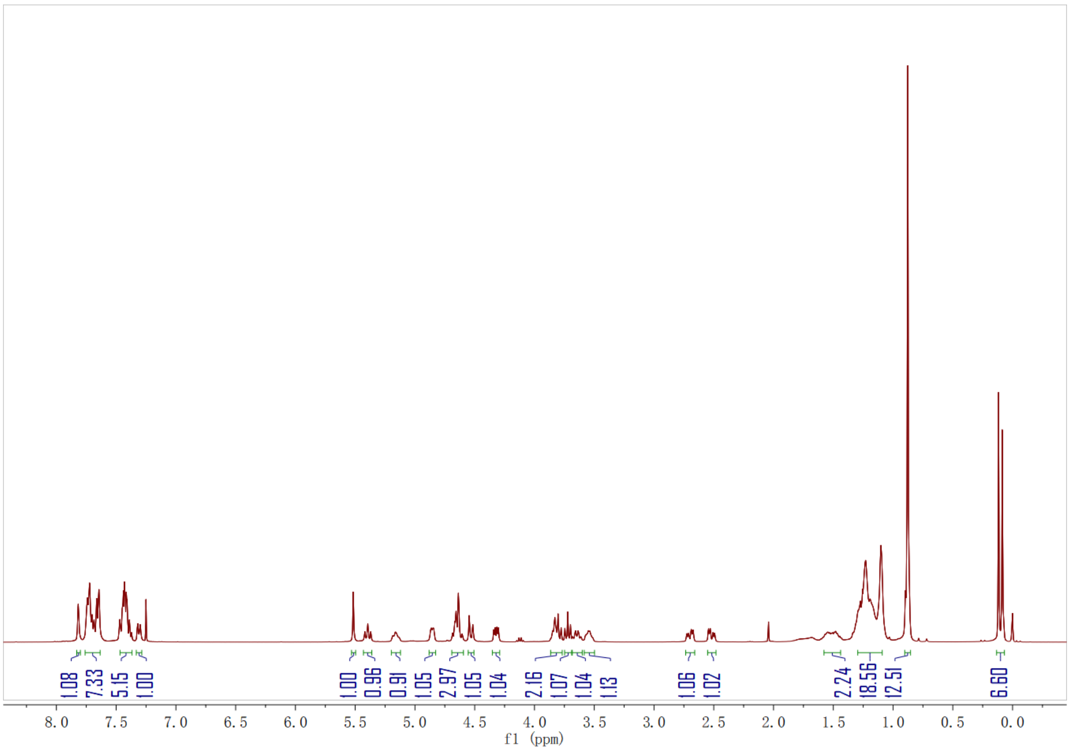

1.08
7.33
5.15
1.00
1.00
0.96
0.91
1.05
2.97
1.05
1.04
2.16
1.07
1.04
1.13
1.06
1.02
2.24
18.56
12.51
6.60
8.0 7.5 7.0 6.5 6.0 5.5 5.0 4.5 4.0 3.5 3.0 2.5 2.0 1.5 1.0 0.5 0.0
f1 (ppm)

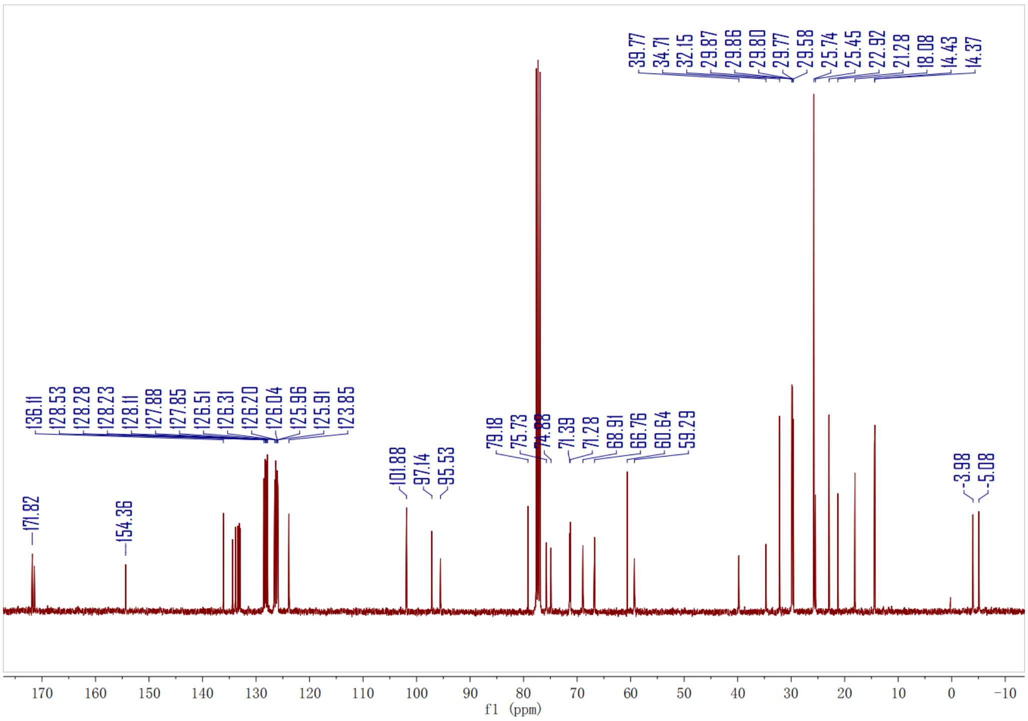

39.77
34.71
32.15
29.87
29.86
29.80
29.77
29.58
25.74
25.45
22.92
21.28
18.08
14.43
14.37
136.11
128.53
128.28
128.23
128.11
127.88
127.85
126.51
126.31
126.20
126.04
125.96
125.91
123.85
79.18
75.73
71.39
71.28
68.91
66.76
60.64
59.29
171.82
154.36
101.88
97.14
95.53
-3.98
-5.08
170 160 150 140 130 120 110 100 90 80 70 60 50 40 30 20 10 0 -10
f1 (ppm)

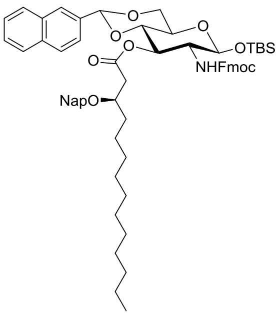
O
O
O
O
OTBS
NHFmoc
O
NapO

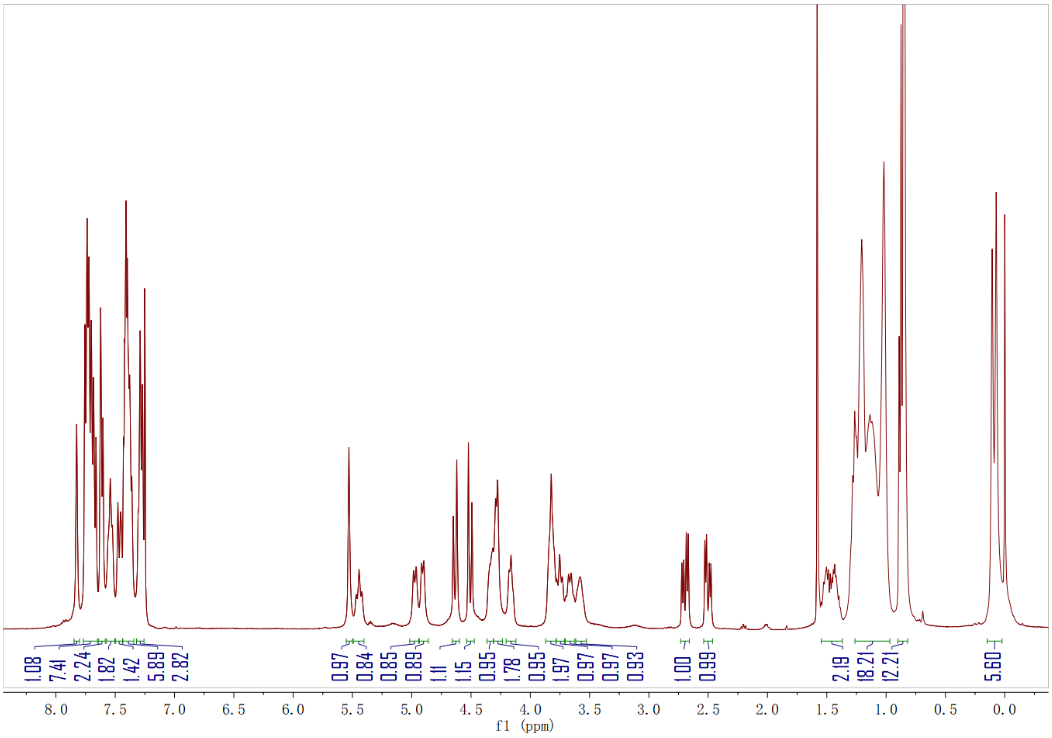
1.08
7.41
2.24
1.82
1.42
5.89
2.82
0.97
0.84
0.85
0.89
1.11
1.15
0.95
1.78
0.95
1.97
0.97
0.97
0.93
1.00
0.99
2.19
18.21
12.21
5.60
8.0 7.5 7.0 6.5 6.0 5.5 5.0 4.5 4.0 3.5 3.0 2.5 2.0 1.5 1.0 0.5 0.0
f1 (ppm)

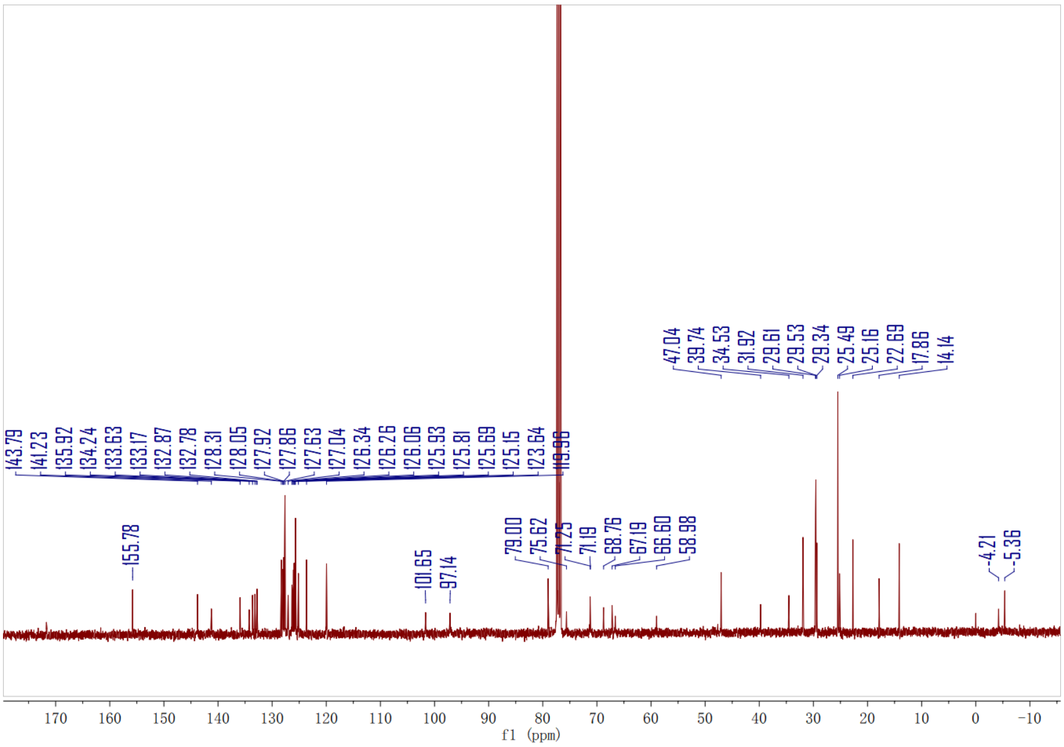
143.79
141.23
135.92
134.24
133.63
133.17
132.87
132.78
128.31
128.05
127.92
127.86
127.63
127.04
126.34
126.26
126.06
125.93
125.81
125.69
125.15
123.64
119.98
155.78
101.65
97.14
79.00
75.62
71.25
71.19
68.76
67.19
66.60
58.98
47.04
39.74
34.53
31.92
29.61
29.53
29.34
25.49
25.16
22.69
17.86
14.14
-4.21
-5.36
170 160 150 140 130 120 110 100 90 80 70 60 50 40 30 20 10 0 -10
f1 (ppm)

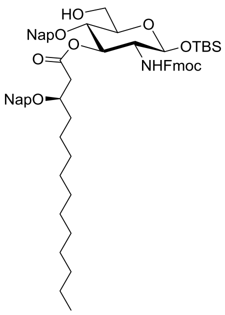

HO
NapO
O
O
OTBS
NHFmoc
O
NapO

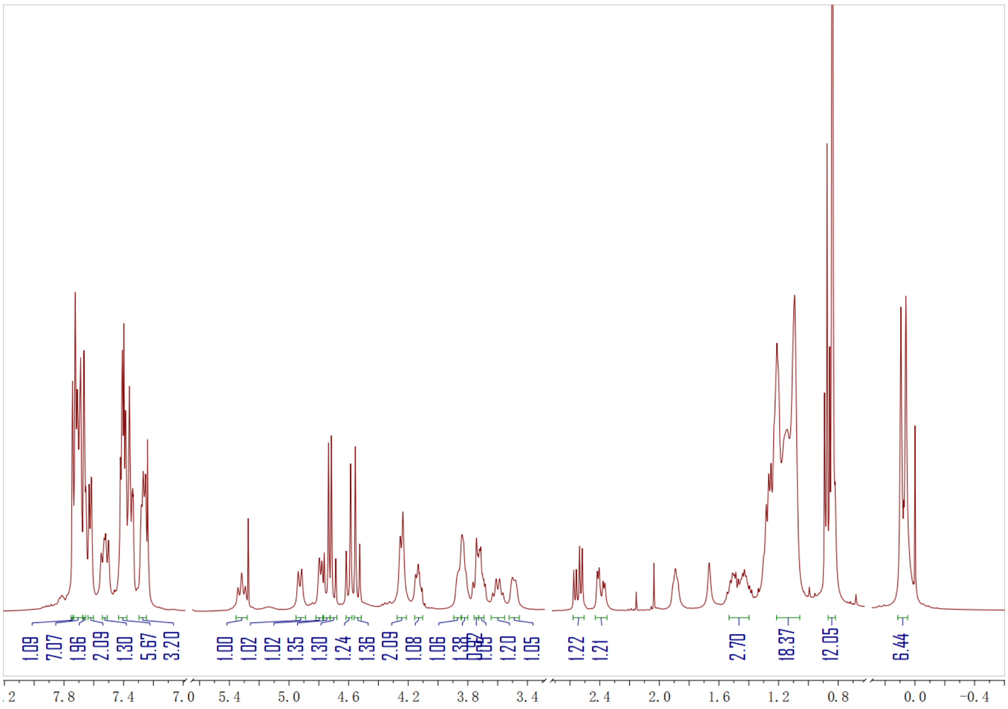

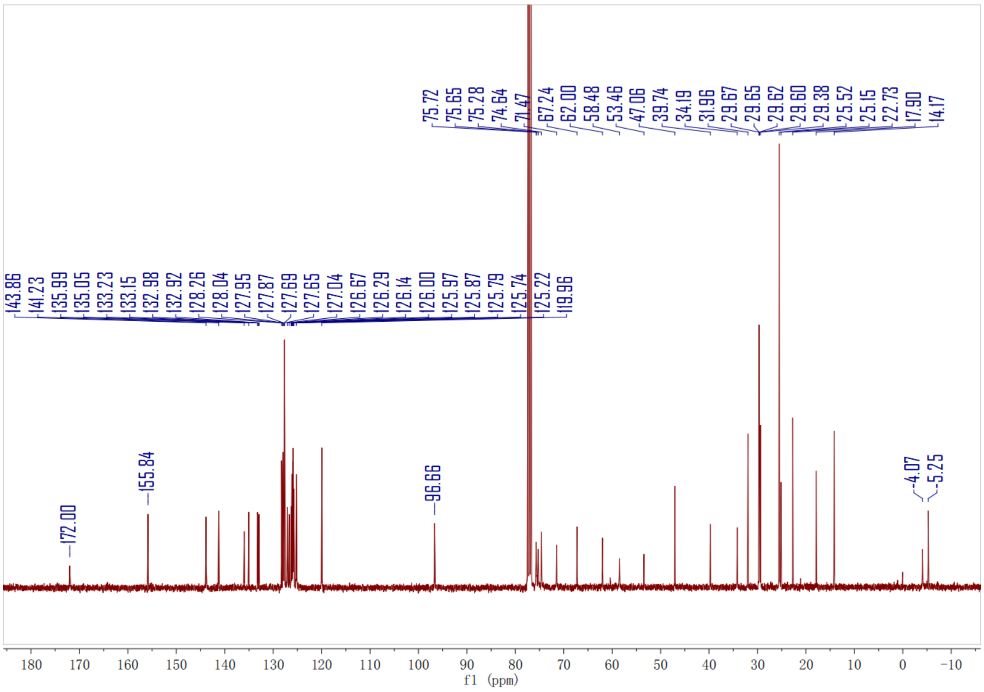

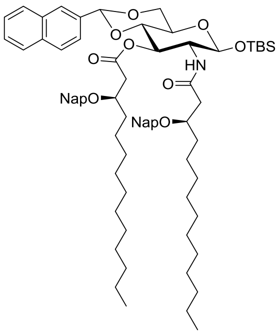

O
O
O
OTBS
O
HN
O
O
NapO
NapO

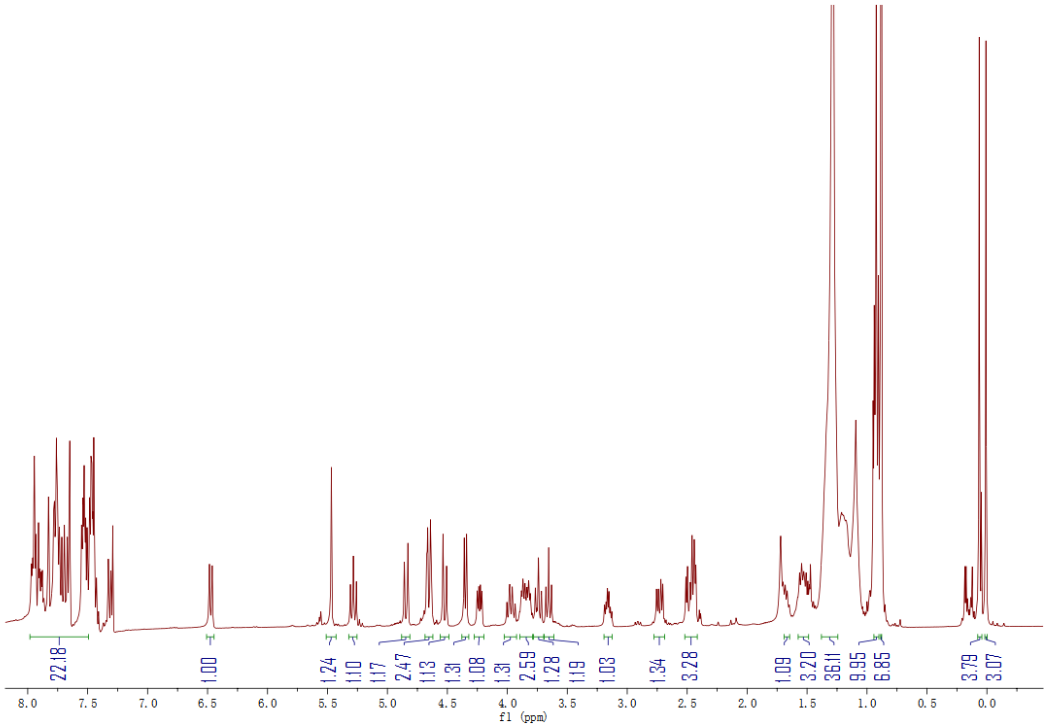

22.18
1.00
1.24
1.10
1.17
2.47
1.13
1.31
1.08
1.31
2.59
1.28
1.19
1.03
1.34
3.28
1.09
3.20
36.11
9.95
6.85
3.79
3.07
8.0 7.5 7.0 6.5 6.0 5.5 5.0 4.5 4.0 3.5 3.0 2.5 2.0 1.5 1.0 0.5 0.0
f1 (ppm)

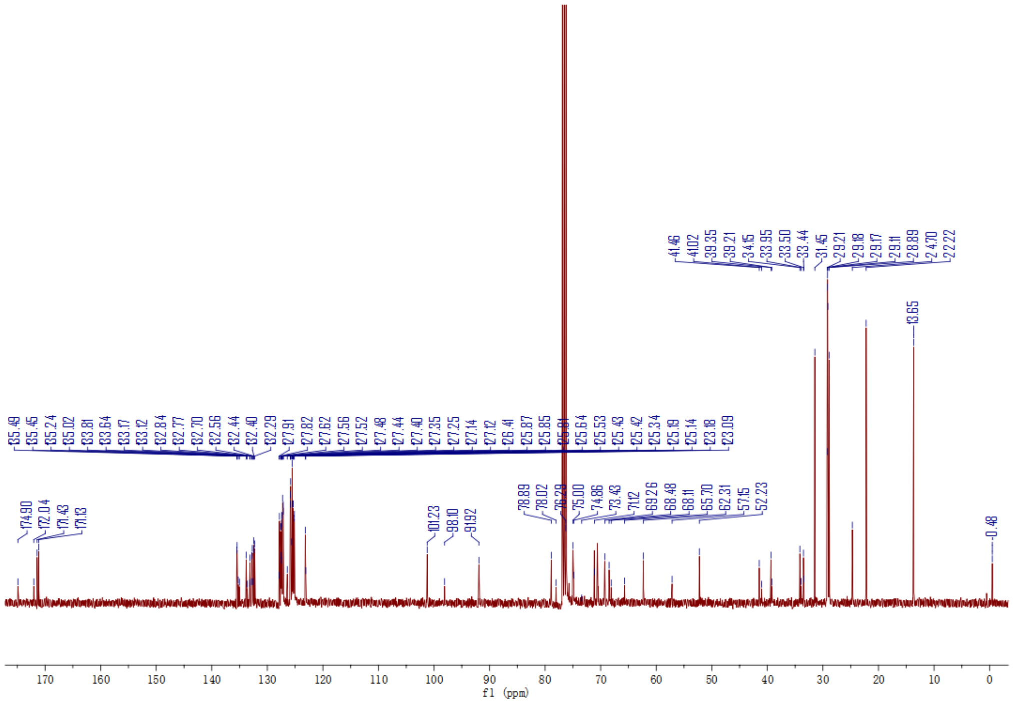

174.90
172.04
171.43
171.13
135.49
135.45
135.24
135.02
133.81
133.64
133.17
133.12
132.84
132.77
132.70
132.56
132.44
132.40
132.29
127.91
127.82
127.62
127.56
127.52
127.48
127.44
127.40
127.35
127.25
127.14
127.12
126.41
125.87
125.85
125.64
125.53
125.43
125.42
125.34
125.19
125.14
123.18
123.09
101.23
98.10
91.92
78.89
78.02
75.00
74.86
73.43
71.12
69.26
68.48
68.11
65.70
62.31
57.15
52.23
41.46
41.02
39.35
39.21
34.15
33.95
33.50
33.44
31.45
29.21
29.18
29.17
29.11
28.89
24.70
22.22
13.65
-0.48
170 160 150 140 130 120 110 100 90 80 70 60 50 40 30 20 10 0
f1 (ppm)

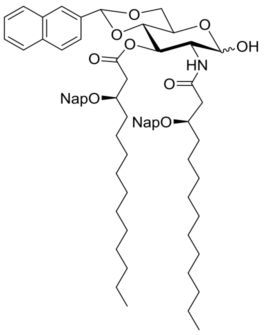
O
O
O
OH
O
HN
O
NapO
NapO

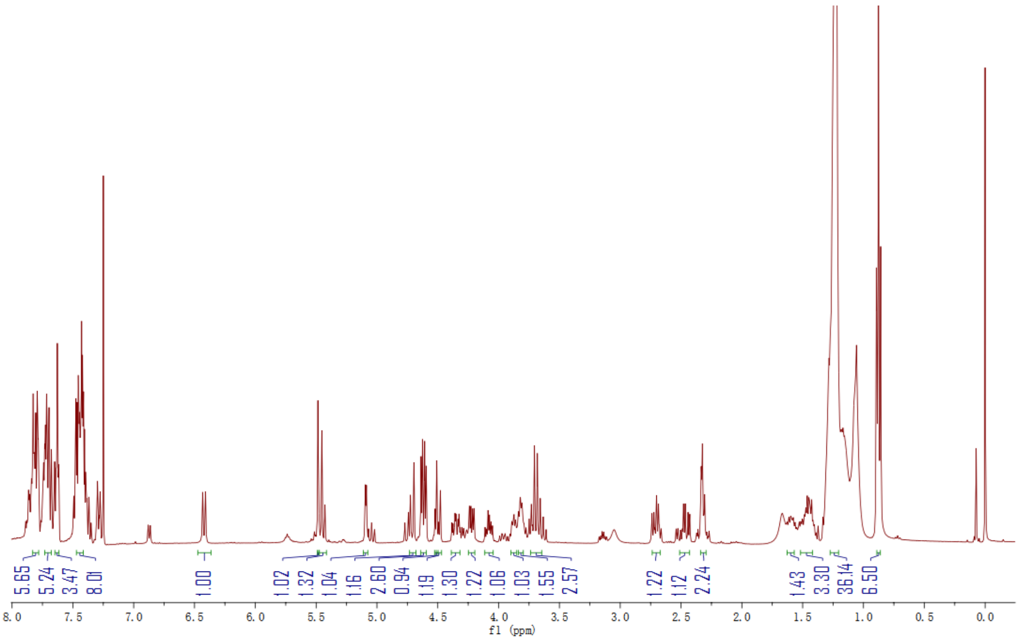
5.65
5.24
3.47
8.01
1.00
1.02
1.32
1.04
1.16
2.60
0.94
1.19
1.30
1.22
1.06
1.03
1.55
2.57
1.22
1.12
2.24
1.43
3.30
36.14
6.50
8.0 7.5 7.0 6.5 6.0 5.5 5.0 4.5 4.0 3.5 3.0 2.5 2.0 1.5 1.0 0.5 0.0
f1 (ppm)

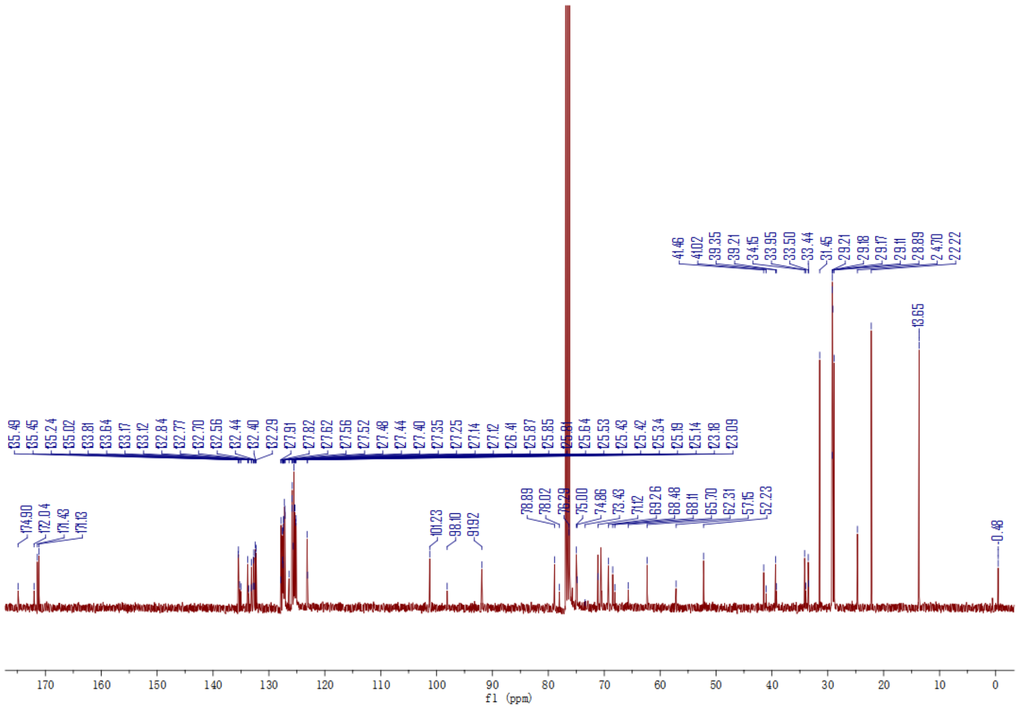
41.46
41.02
39.35
39.21
34.15
33.95
33.50
33.44
31.45
29.21
29.18
29.17
29.11
28.89
24.70
22.22
13.65
135.49
135.45
135.24
135.02
133.81
133.64
133.17
133.12
132.84
132.77
132.70
132.56
132.44
132.40
132.29
127.91
127.82
127.62
127.56
127.52
127.48
127.44
127.40
127.35
127.25
127.14
127.12
126.41
125.87
125.85
125.81
125.64
125.53
125.43
125.42
125.34
125.19
125.14
123.18
123.09
174.90
172.04
171.43
171.13
101.23
98.10
91.92
78.89
78.02
76.29
75.00
74.86
73.43
71.12
69.26
68.48
68.11
65.70
62.31
57.15
52.23
-0.48
170 160 150 140 130 120 110 100 90 80 70 60 50 40 30 20 10 0
f1 (ppm)

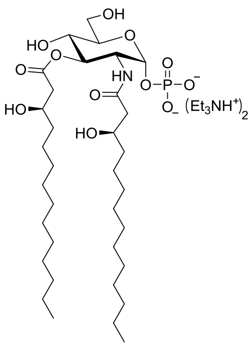
OH
HO
O
O
HN
O
O
O-P-O
O
(Et3NH+)2
HO
HO

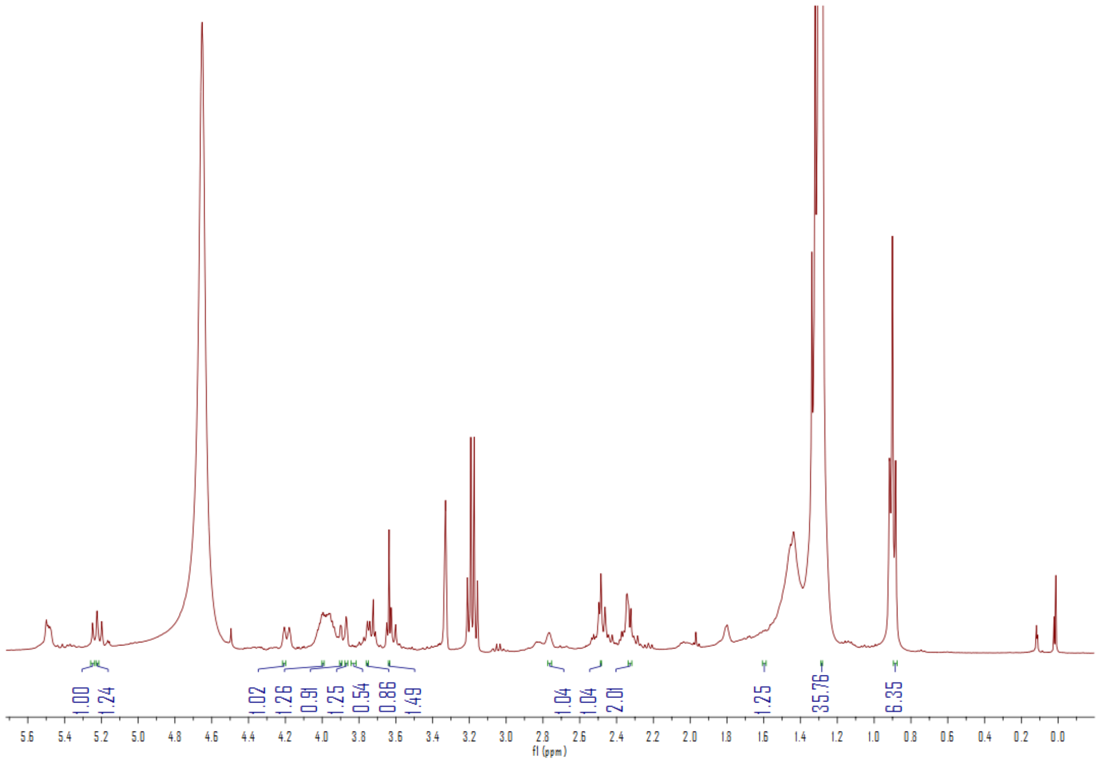
1.00
1.24
1.02
1.26
0.91
1.25
0.54
0.86
1.49
1.04
1.04
2.01
1.25
35.76
6.35
5.6
5.4
5.2
5.0
4.8
4.6
4.4
4.2
4.0
3.8
3.6
3.4
3.2
3.0
2.8
2.6
2.4
2.2
2.0
1.8
1.6
1.4
1.2
1.0
0.8
0.6
0.4
0.2
0.0
f1 (ppm)

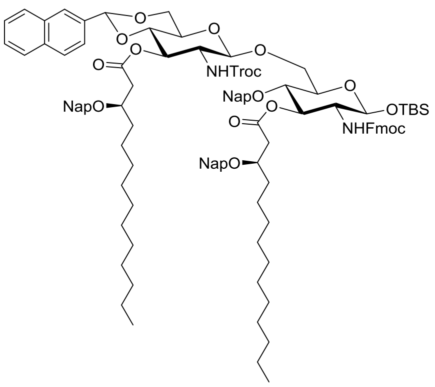
O
O
O
O
NHTroc
O
NapO
O
O
OTBS
NHFmoc
O
NapO
NapO
O
NapO

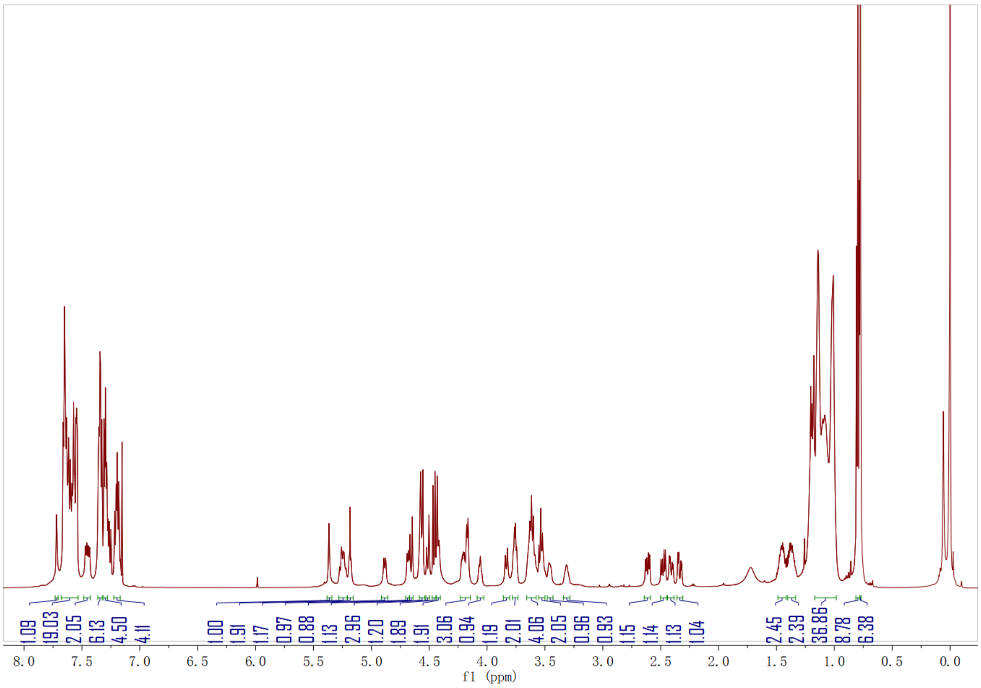
1.09
19.03
2.05
6.13
4.50
4.11
1.00
1.91
1.17
0.97
0.88
1.13
2.96
1.20
1.89
1.91
3.06
0.94
1.19
2.01
4.06
2.05
0.96
0.93
1.15
1.14
1.13
1.04
2.45
2.39
36.86
8.78
6.38
8.0 7.5 7.0 6.5 6.0 5.5 5.0 4.5 4.0 3.5 3.0 2.5 2.0 1.5 1.0 0.5 0.0
f1 (ppm)

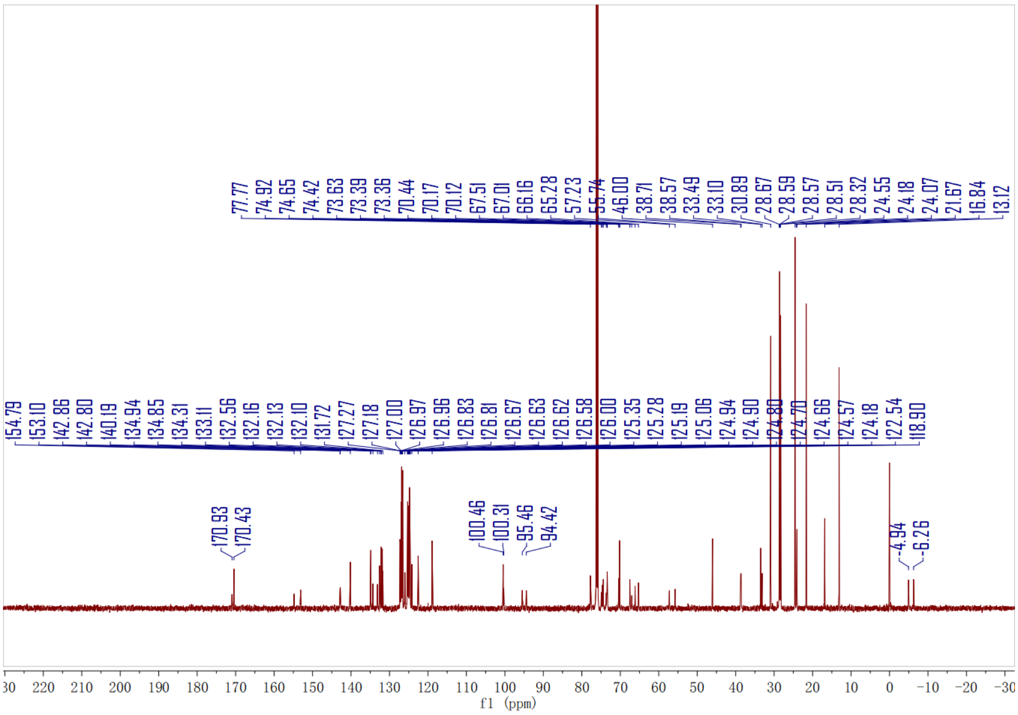
77.77
74.92
74.65
74.42
73.63
73.39
73.36
70.44
70.17
70.12
67.51
67.01
66.16
65.28
57.23
55.74
46.00
38.71
38.57
33.49
33.10
30.89
28.67
28.59
28.57
28.51
28.32
24.55
24.18
24.07
21.67
16.84
13.12
154.79
153.10
142.86
142.80
140.19
134.94
134.85
134.31
133.11
132.56
132.16
132.13
132.10
131.72
127.27
127.18
127.00
126.97
126.96
126.83
126.81
126.67
126.63
126.62
126.58
126.00
125.35
125.28
125.19
125.06
124.94
124.90
124.80
124.70
124.66
124.57
124.18
122.54
118.90
170.93
170.43
100.46
100.31
95.46
94.42
-4.94
-6.26
230 220 210 200 190 180 170 160 150 140 130 120 110 100 90 80 70 60 50 40 30 20 10 0 -10 -20 -30
f1 (ppm)

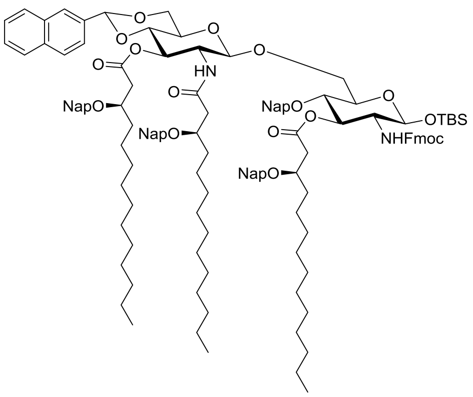

O
O
O
O
HN
O
O
NapO
O
NapO
NapO
O
OTBS
O
NHFmoc
NapO

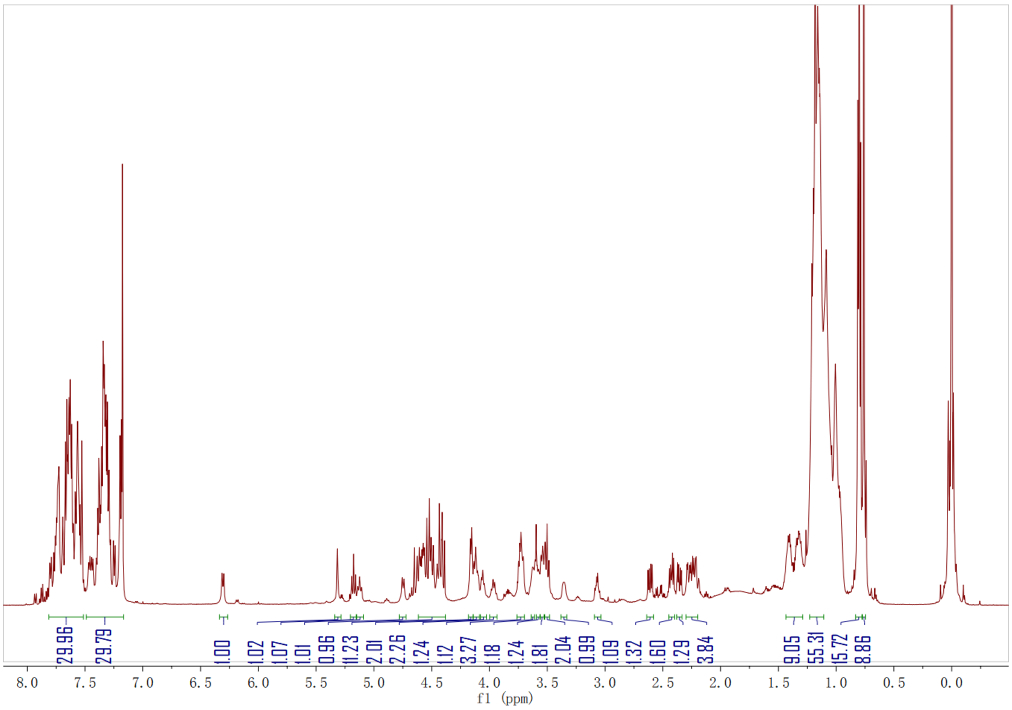

29.96
29.79
1.00
1.02
1.07
1.01
0.96
11.23
2.01
2.26
1.24
1.12
3.27
1.18
1.24
1.81
2.04
0.99
1.09
1.32
1.60
1.29
3.84
9.05
55.31
15.72
8.86
8.0 7.5 7.0 6.5 6.0 5.5 5.0 4.5 4.0 3.5 3.0 2.5 2.0 1.5 1.0 0.5 0.0
f1 (ppm)

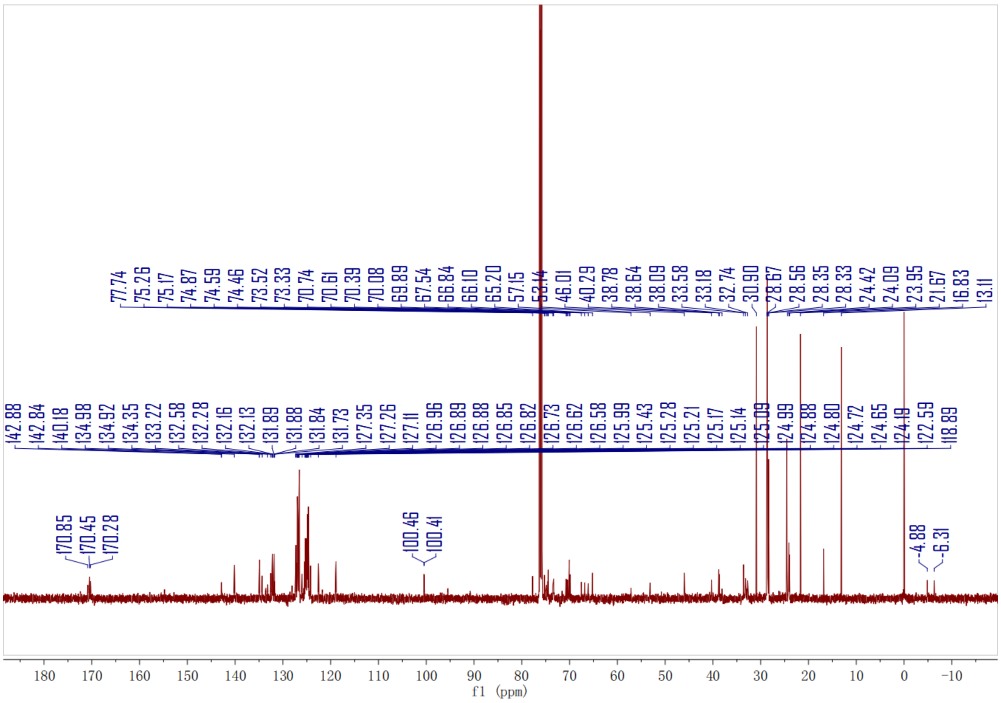

77.74 75.26 75.17 74.87 74.59 74.46 73.52 73.33 70.74 70.61 70.39 70.08 69.89 67.54 66.84 66.10 65.20 57.15 52.14 46.01 40.29 38.78 38.64 38.09 33.58 33.18 32.74 30.90 28.67 28.56 28.35 28.33 24.42 24.09 23.95 21.67 16.83 13.11
142.88 142.84 140.18 134.98 134.92 134.35 133.22 132.58 132.28 132.16 132.13 131.89 131.88 131.84 131.73 127.35 127.26 127.11 126.96 126.89 126.88 126.85 126.82 126.73 126.62 126.58 125.99 125.43 125.28 125.21 125.17 125.14 125.09 124.99 124.88 124.80 124.72 124.65 124.19 122.59 118.89
170.85 170.45 170.28
100.46 100.41
-4.88 -6.31
180 170 160 150 140 130 120 110 100 90 80 70 60 50 40 30 20 10 0 -10
f1 (ppm)

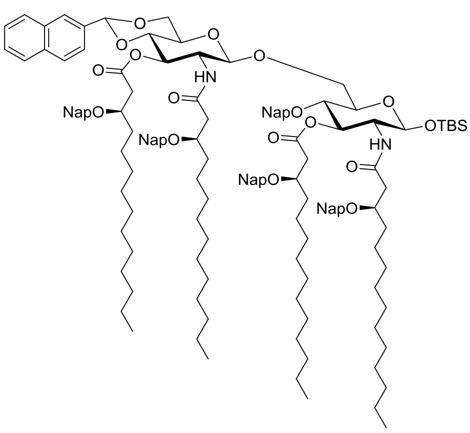

NapO
HN
OTBS

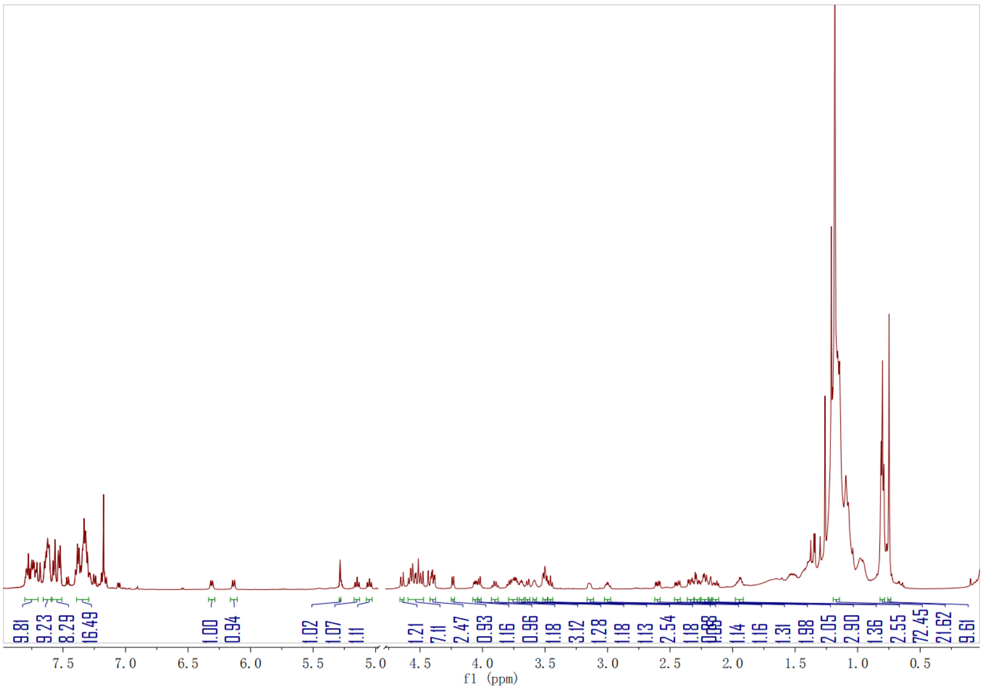

f1 (ppm)

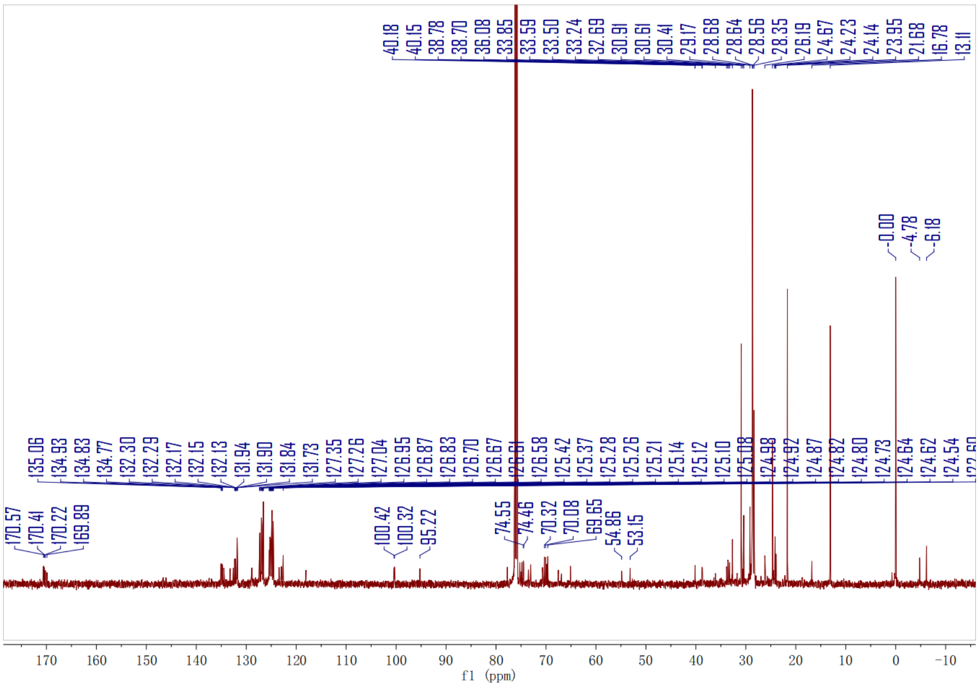

f1 (ppm)

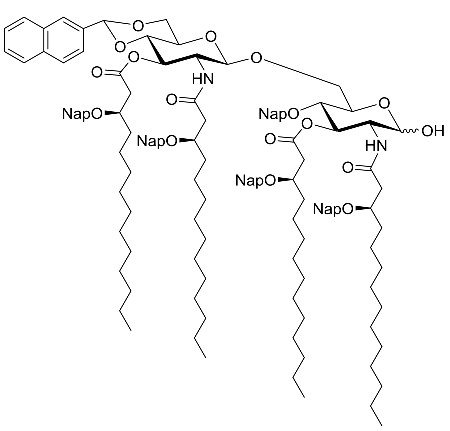

O
O
O
O
O
HN
NapO
NapO
NapO
O
O
HN
OH
NapO
O
NapO
O

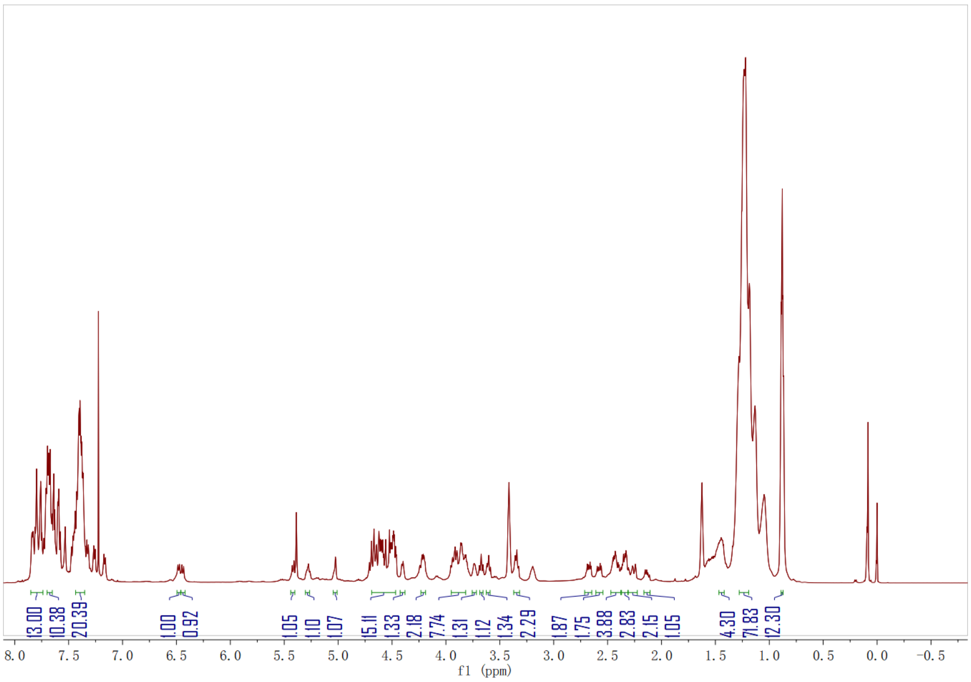

13.00
10.38
20.39
1.00
0.92
1.05
1.10
1.07
15.11
1.33
2.18
7.74
1.31
1.12
1.34
2.29
1.87
1.75
3.88
2.83
2.15
1.05
4.30
71.83
12.30
f1 (ppm)

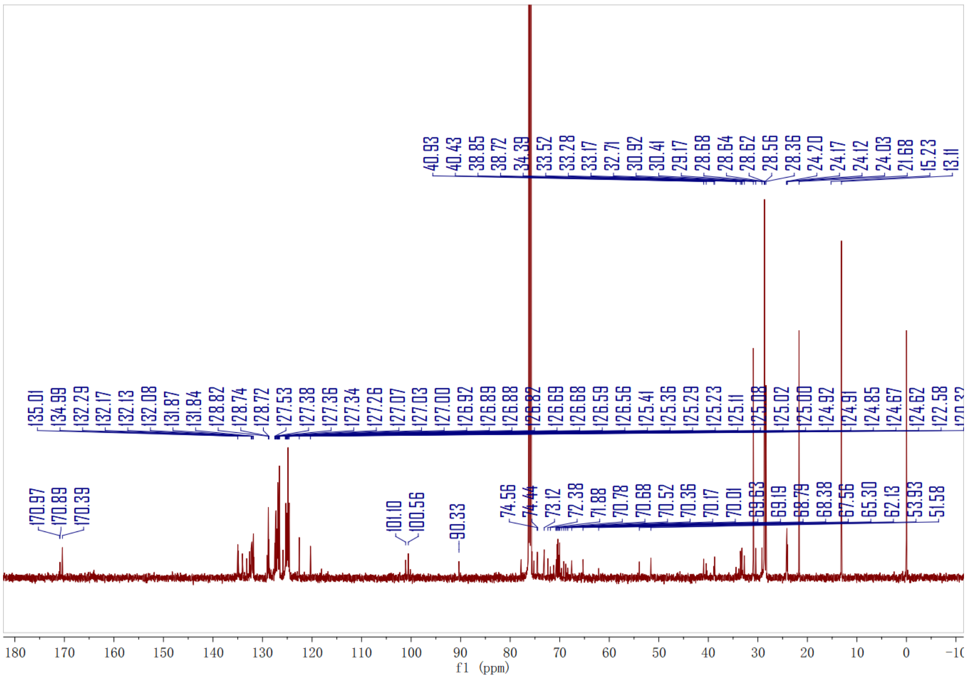

f1 (ppm)

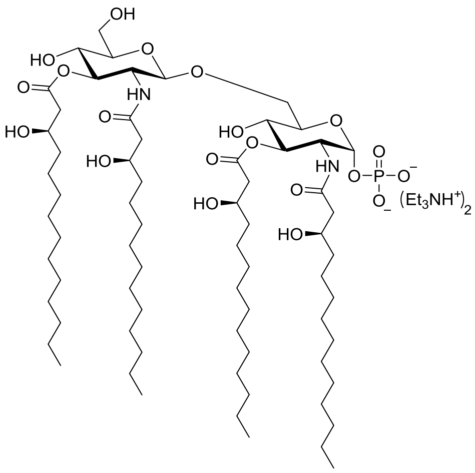

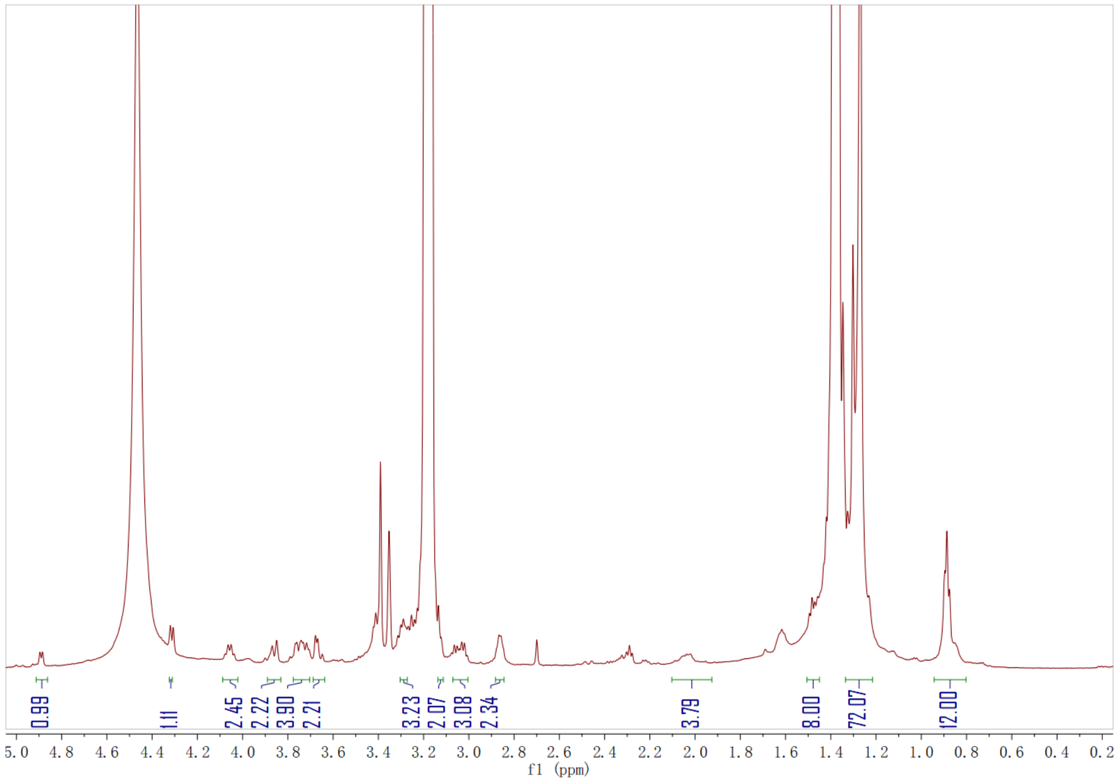
0.99
1.11
2.45
2.22
3.90
2.21
3.23
2.07
3.08
2.34
3.79
8.00
72.07
12.00
5.0 4.8 4.6 4.4 4.2 4.0 3.8 3.6 3.4 3.2 3.0 2.8 2.6 2.4 2.2 2.0 1.8 1.6 1.4 1.2 1.0 0.8 0.6 0.4 0.2
f1 (ppm)
